# A deep survey of alternative splicing in grape reveals changes in the splicing machinery related to tissue, stress condition and genotype

**DOI:** 10.1186/1471-2229-14-99

**Published:** 2014-04-17

**Authors:** Nicola Vitulo, Claudio Forcato, Elisa Corteggiani Carpinelli, Andrea Telatin, Davide Campagna, Michela D'Angelo, Rosanna Zimbello, Massimiliano Corso, Alessandro Vannozzi, Claudio Bonghi, Margherita Lucchin, Giorgio Valle

**Affiliations:** 1 CRIBI Biotechnology Centre, University of Padua, Padua, Italy; 2 Department of Agronomy, Food, Natural resources, Animals and Environment, DAFNAE, University of Padua, Padua, Italy; 3 CIRVE, Centre for Research in Viticulture and Enology, University of Padua, Padua, Italy; 4 Department of Biology, University of Padua, Padua, Italy

**Keywords:** Alternative splicing, Transcriptome, RNAseq, Grapevine

## Abstract

**Background:**

Alternative splicing (AS) significantly enhances transcriptome complexity. It is differentially regulated in a wide variety of cell types and plays a role in several cellular processes. Here we describe a detailed survey of alternative splicing in grape based on 124 SOLiD RNAseq analyses from different tissues, stress conditions and genotypes.

**Results:**

We used the RNAseq data to update the existing grape gene prediction with 2,258 new coding genes and 3,336 putative long non-coding RNAs. Several gene structures have been improved and alternative splicing was described for about 30% of the genes. A link between AS and miRNAs was shown in 139 genes where we found that AS affects the miRNA target site. A quantitative analysis of the isoforms indicated that most of the spliced genes have one major isoform and tend to simultaneously co-express a low number of isoforms, typically two, with intron retention being the most frequent alternative splicing event.

**Conclusions:**

As described in *Arabidopsis*, also grape displays a marked AS tissue-specificity, while stress conditions produce splicing changes to a minor extent. Surprisingly, some distinctive splicing features were also observed between genotypes. This was further supported by the observation that the panel of Serine/Arginine-rich splicing factors show a few, but very marked differences between genotypes. The finding that a part the splicing machinery can change in closely related organisms can lead to some interesting hypotheses for evolutionary adaptation, that could be particularly relevant in the response to sudden and strong selective pressures.

## Background

Several reasons make grapevine particularly interesting: it is the most cultivated fruit plant covering approximately 7.5 million hectares in 2012 (http://www.oiv.int), with a long history of domestication, as well as a useful model organism since it seems to have maintained the ancestral genomic structure of the primordial flowering plants. The complete genome sequence was obtained in 2007 by two independent projects [1,2]. The availability of the genomic sequence gave the opportunity to conduct several genome-wide studies focused on different aspects of grape biology such as berry development and response to different biotic and abiotic stresses [3-10].

However the eukaryotic transcriptome, and in particular the plant transcriptome, is far more complex than previously believed, alternative splicing and non coding transcripts being amongst the major causes contributing to this complexity. Recent works pointed out the extensive diffusion of these phenomena in plants and their importance in gene expression and stress response [11-14].

Alternative splicing (AS) is one of the main mechanisms that forge transcriptome plasticity and proteome diversity [15]. Different studies based on computational analysis on both expressed sequence tags and high-throughput RNA sequencing provide an estimate of the frequency of these events. For example, 20–30% of transcripts were found to be alternatively spliced in both *Arabidopsis thaliana* and rice (*Oryza sativa*) by employing large-scale EST-genome alignments [15,16]. Recently, deep sequencing of the transcriptome using high-throughput RNA sequencing (RNAseq) increased this estimate showing that more that 60% of intron-containing genes in *Arabidopsis* are alternatively spliced [12]. Although most AS events of plants have not yet been characterized, there is a strong evidence indicating that they are spatially and developmentally regulated, playing important roles in many plant functions such as stress response [17]. Moreover, since AS events are different at intraspecific level in several plant species, it was suggested that they may be correlated with niche specialization resulting from domestication in different geographical regions [18,19].

Recently, the human cell transcriptional landscape was extensively investigated by the Encode Project [20] revealing that most genes tend to express several isoforms at the same time, with one isoform being predominant across different cell types. Moreover a recent study confirmed these observations, showing that for 80% of the expressed genes in primary tissue cultures, the major transcript is expressed at a considerably higher level (at least twice) than any other isoform [21]. Similar extensive studies are still missing in plants.

Some emerging evidence indicates that a large fraction of the eukaryotic genome is transcribed [22-24] and that a considerable amount of the transcriptome is composed by non-coding RNA (ncRNA) that may play a key role as a regulator in many cellular processes. A poorly characterized class of plant ncRNA is composed of long non-coding RNA (lncRNAs), mRNA-like transcripts greater than 200 bases transcribed by RNA polymerase II, polyadenylated, spliced and mostly localized in the nucleus [25]. In plants a systematic identification of long non coding transcripts has only been done for a few species [13,14,26,27]. In *Arabidopsis* for example, using a tailing-array based method Liu *et al.* identified 6480 long intergenic non-coding transcripts, 2708 of which were confirmed by RNA sequencing experiments [13]. Based on their characteristics, lncRNAs can be classified as natural antisense transcripts (NATs), long intronic noncoding RNAs and long intergenic noncoding RNAs (lincRNAs). Some of these transcripts have been shown to be involved in important biological processes such as developmental regulation and stress response, although the detailed mechanisms by which they operate are mostly unknown [25]. Moreover, several lncRNAs were found to be involved in plant reproductive development [28] and responses to pathogen invasion [13,14]. Furthermore it has been observed both in plant [13,14] and in vertebrate [29,30] that lncRNAs have both tissue and temporal-dependent expression patterns.

The extent and complexity of the transcriptional landscape in plants is not yet well characterized. Recent advances in high-throughput DNA sequencing technologies applied to transcriptome analyses have opened new and exciting possibilities of investigation [31]. RNAseq has been successfully applied in several studies including gene prediction improvement [32,33], isoform identification [11,12,34], isoform quantification [35,36], non-coding transcript discovery [29,30,37].

Here we present a deep survey on the grape transcriptome, based on 124 RNAseq SOLiD libraries from leaf, root and berry, from different genotypes under different physiological and stress conditions.

The high coverage of our samples allowed us to review the *Vitis vinifera* gene annotation and to extend it to include alternative spliced isoforms. The impact of alternative splicing on miRNA target sites was also investigated. Our data showed that alternative splicing is correlated to tissue as well as genotypes. Finally, we developed a stringent pipeline to identify long non-coding RNAs, that were annotated based on their expression in different tissues and stress conditions.

## Results and discussion

### Dataset

RNAseq data came from a parallel work (paper in preparation) aiming to study the response to water-deficiency and salt stresses of two rootstocks, the widely used 101.14 and the experimental M4, kindly provided by prof. A. Scienza, University of Milan (Italy). The commercial rootstock 101.14 was derived from a cross of *V. riparia* x *V. rupestris*, while M4 is an experimental rootstock derived from a cross of (*V. vinifera* x *V. berlandieri*) x *V. berlandieri* cv. Resseguier n.1 [38]. It should be noticed that although *V. vinifera, V. riparia, V. rupestris and V. berlandieri* are generally classified as 4 different species, they are all able to cross fertilize and to produce fertile progenies; therefore, they are strongly related and should be considered as the same biological species. As a background work of the project (data not shown) the two rootstock genomes were resequenced. We found that the average frequency of single nucleotide variants is about 1/200 bases, very similar to what is found when comparing different *V. vinifera* cultivars. Excluding possible gene family expansions, no private genes were found in the rootstock genotypes. This further supports the idea that we are working on the same biological species. In any case, the aim of this work was not the annotation of a *Vitis* “pangenome”, but the improvement of the *Vitis vinifera* reference genome.

Some RNAseq analyses were also performed on Cabernet Sauvignon, that is a well known cultivar of *V. vinifera*. More details can be found in the materials and methods section. A total of 124 samples from leaves, roots and berries were sequenced using SOLiD technology producing approximately 6 billion of directional 75/35 bases paired-end RNAseq reads.

### Improvement of the grape gene prediction

The grape gene prediction and annotation (http://genomes.cribi.unipd.it/grape), available before the present work is referred to as v1 and followed the v0 annotation soon after the release of the grape PN40024 genomic sequence [1]. The v1 annotation improved to some extent the previous annotation and now it represents the generally used gene reference of grape. The new potential of RNAseq technology is now revealing some weaknesses of the v1 annotation and at the same time offering the opportunity for a through and systematic study of the grape transcriptome.

Two recent works raised some concern about the v1 annotation. Firstly, a comparison between v1 and v0 showed that 6,089 genes annotated in v0 were not present in v1 [39]. Although some of those genes may be artefacts, others are certainly genuine grape genes and should be reintegrated into the annotation. Secondly, the v1 annotation did not attempt to describe alternative isoforms. This was pointed out by a *de novo* transcriptome assembly of RNAseq of *V. vinifera* cv. Corvina, that allowed the identification of 19,517 splice isoforms among 9,463 known genes and 2,321 potentially novel protein coding genes [4].

Motivated by these observations we improved and updated the grape gene prediction, integrating the information derived from the considerable amount of newly available data and setting up rigorous bioinformatic procedures based on several filtering steps, to limit the number of artifactual genes.

**Figure 1:**
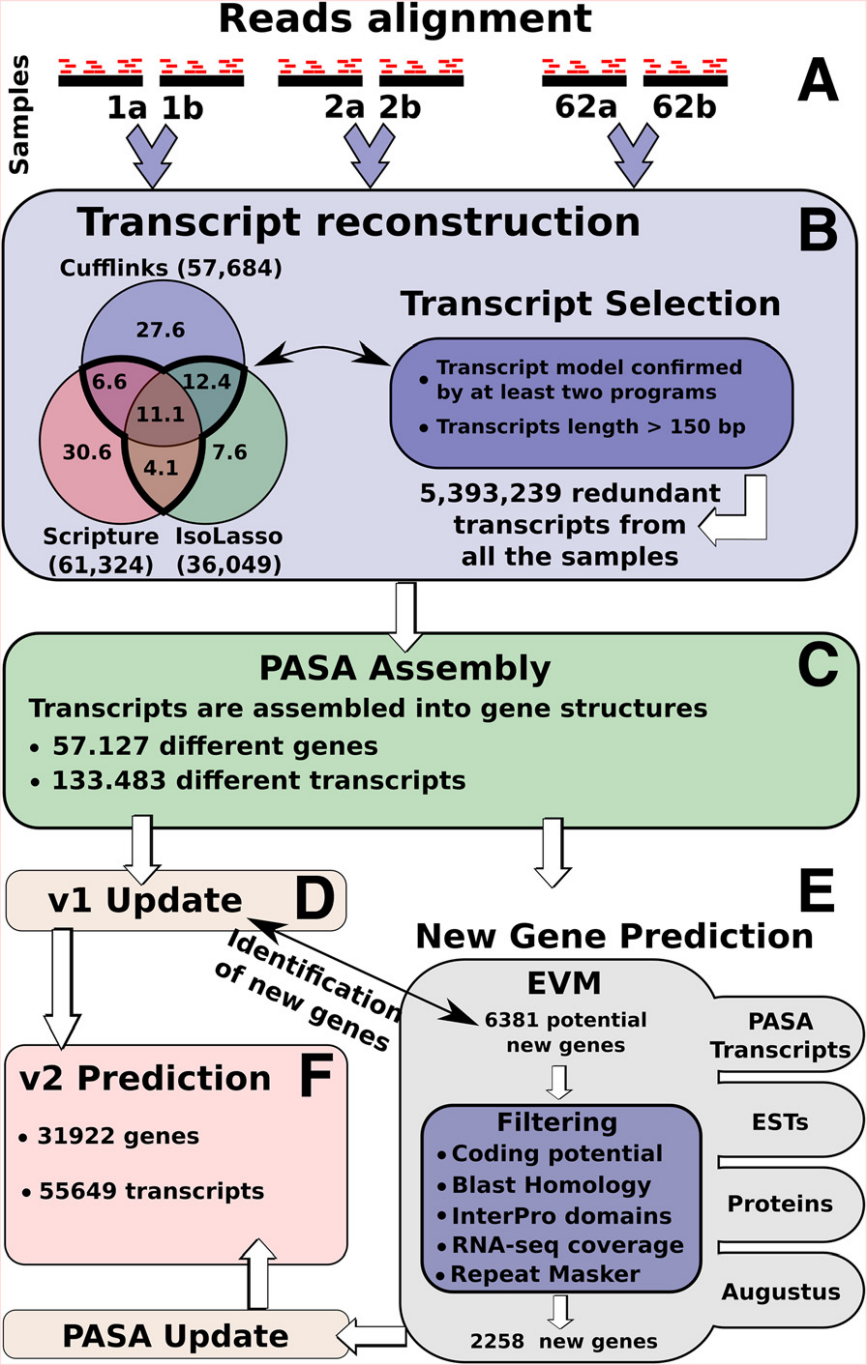
**Gene prediction workflow. (A)** RNAseq samples are aligned on the reference genome. **(B)** Biological replicate alignments are merged together into 64 different datasets. Transcript reconstruction was performed independently on each dataset using three different programs: Cufflinks, Scripture and Isolasso. The Venn diagram shows the percentage of reconstructed transcripts in common among the three software while the numbers between brackets indicates the average number of reconstructed transcripts per sample. We selected only those transcript models predicted by at least two programs and with a length higher than 150 bases. **(C)** The selected transcripts were assembled using PASA software. **(D)** PASA assemblies were used to update v1 gene predictions. **(E)** A new gene prediction was performed integrating with EvidenceModeler (EVM) software different sources of evidence such as PASA transcripts, ESTs and proteins alignments and Augustus prediction trained with PASA assemblies. The produced gene set was compared to v1 gene prediction and only the new gene loci were selected for further analysis. After applying different filtering criteria, we obtained a final dataset of 2,258 new genes. **(F)** The final v2 gene prediction integrates genes generated by the steps described in **D** (v1 update) and **E** (new gene prediction).

A detailed workflow describing the different steps of the analysis is presented in Figure 1. The general analysis of the RNAseq data was based on the “align-then-assemble” strategy. Firstly, the RNAseq reads from 124 libraries were aligned onto the reference grape genome using PASS [40]. Then the spliced reads that were not sufficiently supported were discarded as described in the Methods section. Secondly, we used three different software to reconstruct the transcripts: Cufflinks [36], Isolasso [41] and Scripture [37]. Since the 124 RNAseq libraries corresponded to 62 different replicated samples, we merged together the alignments from each replica, thus obtaining 62 datasets. We obtained an average number per dataset of 57,000, 36,000 and 61,000 reconstructed transcripts respectively for Cufflinks, Isolasso and Scripture (Figure 1, panel B). Finally, in order to reduce the number of misassembled transcripts and artefacts, we removed all the assemblies that were not predicted by at least two of the three programs. To reconstruct the transcripts, all the datasets were clustered with PASA [42] producing 133,483 individual isoforms, belonging to 57,127 genes (Figure 1, panel C).

The gene prediction was performed in two different steps. Firstly, we updated the v1 gene prediction incorporating the RNAseq reconstructed transcripts using the PASA software [42] (Figure 1, panel D). PASA is a tool designed to model and update gene structures using alignment evidence and it is able to correct exon boundaries, add UTRs and model for alternative splicing. Secondly, we performed a new gene prediction integrating evidence from ESTs, proteins and RNAseq (Figure 1, panel E, see Methods). This second step identified 2,258 new genes, 80% of which were found to have at least one gene ontology annotation (see Methods, Additional file 1). Gene enrichment analysis revealed that the addition of these new genes endowed the list of functional categories with functions that were previously under-represented (Additional file 2: Figure S1). Among the most significant categories, we found terms related to nucleotide binding site such as “ADP binding”, “adenyl ribonucleotide binding” or “purine ribonucleotide binding”. Interestingly, most genes associated with this domain are annotated as “disease resistance” [43].

The new gene prediction, called v2, contains 31,922 genes and 55,649 transcripts (Figure 1, panel F). The v2 gene prediction showed several differences from the previous prediction, such as longer transcripts and coding sequences (CDS) and a higher number of exons per gene. As reported in Table 1, the incorporation of the RNAseq information led to an important improvement in the prediction of the untranslated regions (UTRs). The v1 UTRs prediction was based on EST data and suffered from the lack of information at the 5′ and 3′ end of transcripts, due to the scarce yield of full length cDNAs in the EST data. RNAseq data provided a decisive contribution to overcome this problem. We found that in the v2 gene prediction the number of genes with a 5′ and 3′ UTRs rose respectively, from 17,082 to 21, 892 and from 20,087 to 23,337. Moreover, we found that the average UTR length of v2 is twofold longer than v1 (Table 1).

**Table 1:** Gene prediction statistics and comparison

	**v1**	**v2**
# genes	29970	31922
# transcripts	29970	55649
# transcripts x gene	1	1,7
Average length	5134	5267
Median length	2741	2917
	**Transcript**	
Average length	1096	1207
Median length	876	990
	**CDS**	
Counts	142332	158834
Average coding length	231	247
Median coding length	129	136
	**Exon**	
Counts	147805	180493
Avg length	270	410
Median length	147	201
Avg exon x gene	4,7	5,3
	**UTR3**	
Counts	22275	43542
Avg length	211	495
Median length	186	356
	**UTR5**	
Counts	20025	42291
Avg length	119	285
Median length	72	176
	**Intron**	
Counts	117835	124393
Avg length	968	1005
Median length	248	263

To evaluate the quality of the exon/intron splicing sites we performed a comparison between v1 and v2 gene predictions and we found that almost 97% of the v1 introns are predicted also in v2. To further asses the quality of the two predictions, we used three different sources of evidence, proteins, ESTs and RNAseq, and we were able to confirm 92% of the shared introns. Interestingly, the analysis showed that almost 29% of the introns are supported by at least two different independent sources of evidence while this number rose to 58% when we considered all the three sources, demonstrating the high quality of the exon/intron boundaries of both gene predictions. More details are available in Additional file 2: Table S1 and S2. When we analysed the splicing sites exclusive to one or the other gene prediction, we were able to confirm only 46% of v1 introns against 85% of the introns confirmed in the v2. As expected, we found that the majority of these exclusive splicing sites are confirmed only by one evidence. In particular we found that the major contribution to the v2 exclusive splicing site is given by the RNAseq data.

As described above, the v2 prediction was generated from v1 using the PASA software, without further manual revision. We observed that v1 and v2 are very similar; however we found 249 v2 genes derived from the fusion of 520 v1 genes, while 183 v2 genes were derived from the splitting of 91 v1 genes (Additional file 3). To discriminate between false/positive fusion/splitting events, we performed a similarity search of each group of fused/split proteins against the Arabidopsis proteome (TAIR10). We evaluated the number and the consistency of the best hit to determine the reliability of the fusion/splitting events (see Methods). We found that of the 249 fused genes, 161 find a better match on v2, while 54 on v1. Whereas of the 91 split genes, 38 have a better match on v2 and 29 on v1 (Additional file 2: Table S3).

A further comparison between v1 and v2 showed that 4,966 genes have a different coding sequence in the two predictions. For each pair of alternative prediction we performed a global pairwise alignment using the Arabidopsis homologous protein as reference. The results show that the majority of v2 genes have a higher score than v1, suggesting that they have a better gene structure (Additional file 2: Table S4 and Figure S2).

Finally we performed a comparison at functional level using InterProScan annotation. We were able to annotate 23,569 v1 genes with at least one InterPro domain, while this number rose to 25,880 genes when we considered v2 gene prediction. As reported in Additional file 2: Figure S3, v2 is better both in terms of number of domains identified and number of annotated genes.

### Alternative splicing prediction and analysis

**Figure 2:**
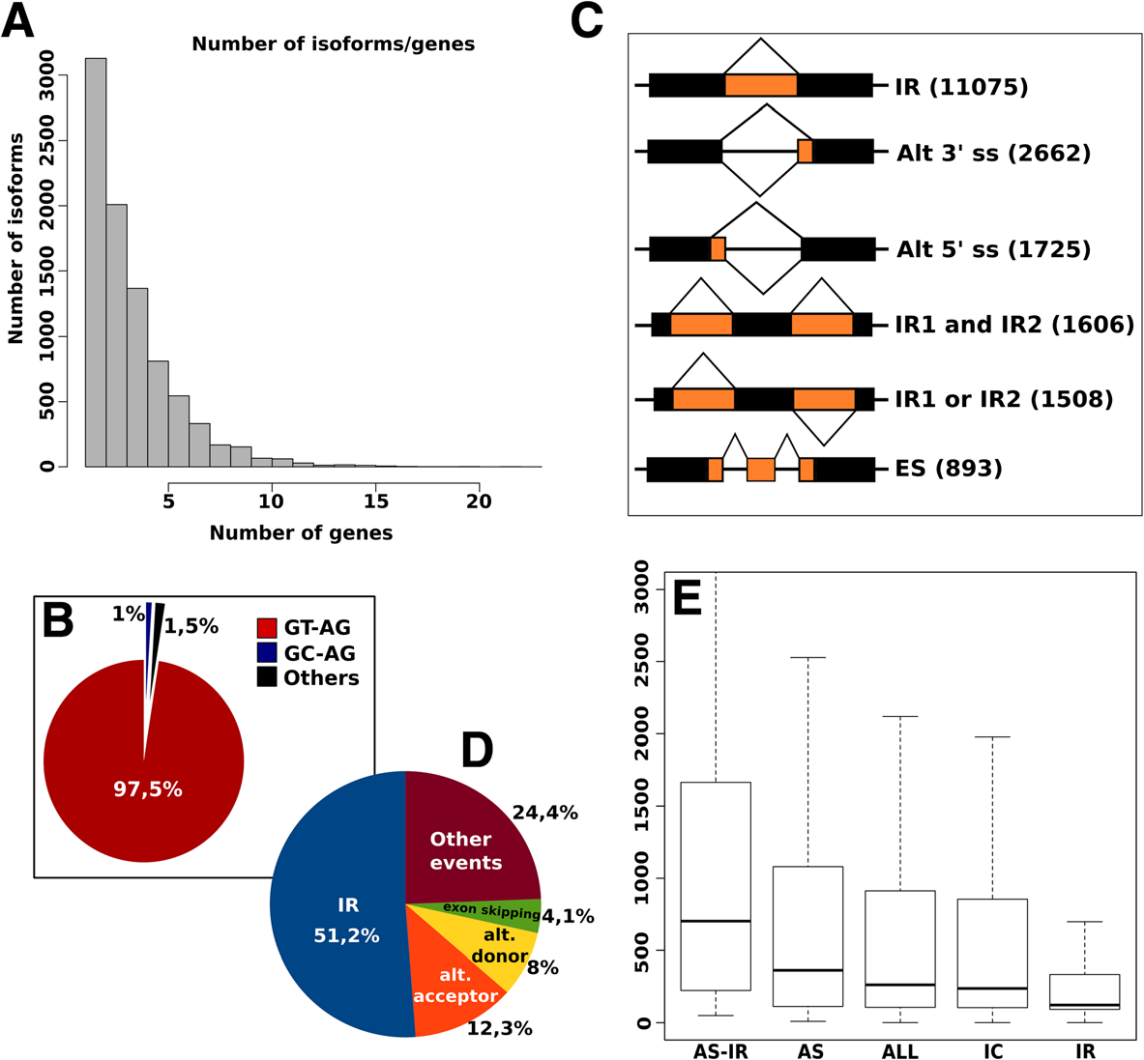
**Alternative splicing analysis. (A)** Number of isoforms per gene distribution. **(B)** Donor and acceptor splicing site distributions. **(C)** Schematic representation of the most frequent splicing event identified in the v2 prediction: intron retention (IR), alternative 3*'* splicing site (Alt 3*'* ss), alternative 5*'* splicing site (Alt 5*'*), exon skipping (ES). The number of events is reported between brackets. **(D)** Pie chart showing the percentage distribution of alternative splicing events. **(E)** Intron size box plot distribution: all introns (ALL), constitutive introns (IC), alternatively spliced introns (ASI), introns that underwent intron retention events (IR), alternatively spliced introns without IR (AS-IR).

We observed that 90% of v2 predicted genes (29,150) contain two or more exons, and 30% (8,668) of these undergo alternative splicing producing 32,395 different isoforms. We also found that 64% of the alternative spliced genes produced more than two isoforms (Figure 2, panel A). Analysis of the acceptor-donor sites shows that 97.5% are canonical GT-AG pairs, while 1% are GC-AG and 1.5% a combination of less frequent non canonical sites. (Figure 2, panel B).

We used ASTALAVISTA [44] to identify and classify the different types of alternative splicing. We identified 21,632 alternative splicing events, affecting 17% of all the introns, distributed into five main categories: intron retention, exon skipping, alternative donor, alternative acceptor and complex events (Figure 2, panel C and D). We found that the most common event is intron retention, involving 77% of the alternatively spliced genes. This AS category is mainly represented by transcripts in which a single intron is optionally included and occurred in 51% of the AS events. On the contrary, exon skipping occurred only in 4.1% of the cases. Moreover we found that the use of alternative acceptors (12.3%) is more frequent than the use of alternative donors (8%). These results are consistent with other studies [11,12,15,34] supporting the idea that intron retention is a common event in plants. In Figure 2 panel E, we compared the size distribution of the retained introns (IR) with that of the total introns (ALL), the constitutive introns (IC), the alternatively spliced introns (ASI) and the alternative splicing events excluding the intron retention events (AS-IR). We found that the size distribution of retained introns is considerably smaller than the intron size of other AS (IR median of 123, AS-IR median of 702), supporting the hypothesis that intron retention is related to intron size [12,34].

We also performed an analysis to identify which gene regions preferentially undergo alternative splicing. We found that about 70% of all AS events occur at the protein-coding level, while 18% and 11% occurred respectively, at the 5′ UTR and 3′ UTR regions. The remaining 1% of the AS events occurred between a coding sequence and a UTR. These values compared reasonably well with the extension of coding sequences (65%), 5′UTRs (17%) and 3′UTRs (18%), indicating that all regions of the transcript are susceptible to alternative splicing without any significant preference. The findings that alternative splicing may not be limited to the sole production of protein diversity also emerged from *Arabidopsis*[45]*.* Moreover of all the genes with at least one isoform, 46% have alternative start sites, while 60% have alternative stop codons.

### Alternative splicing affects miRNA target sites

Unlike animal miRNAs that usually recognize their target on the 3′UTR region, plant miRNAs do not show preferences in terms of target position [46]. To evaluate the impact of the v2 gene prediction on miRNA target prediction, we performed a target analysis using the psRNATarget server [47]. As reported in Table 2, the v2 prediction shows an increased number of miRNA target sites allowing the identification of targets for 13 more miRNAs and 167 new target genes. Interestingly, more that 79% of the target sites in the v1 prediction were identified on coding sequences, while in v2 those were only 55%. On the other hand we found that target regions on 3′ UTRs and 5′ UTRs increased from 11% to 27% and from 9% to 18% respectively on v2 compared to v1, reassessing the importance of UTR regions in plant miRNA target identification (Additional file 4).

**Table 2:** miRNA target site prediction results

	**v1**	**v2**
**# mirna**	168	181
**# genes**	432	599
**# transcripts**	432	1184
**# 3′ UTR**	49	317
**# 5′ UTR**	41	218
**# CDS**	343	658

We investigated the effect of alternative splicing on miRNA target sites. A recent analysis of *Arabidopsis*[48] revealed that mRNA splicing seems to be a possible mechanism to control miRNA-mediated gene regulation. Indeed, alternative splicing could produce different isoforms which may or may not contain functional binding sites, playing an important role in modulating the interaction between miRNA and target.

To test this hypothesis, for each gene with alternative splicing we checked if the target sites were predicted to be present across all the isoforms. We found 286 cases, involving 131 miRNA and 139 genes (23% of the identified miRNA target genes), in which a miRNA binding site is missing in one or more isoforms. Our analysis revealed that in 43% of the cases the missing binding site is the result of a differential mRNA initiation or termination. 54% of the remaining events occurred at the 5′UTR, 21% at the 3′UTR and 23% at the coding sequence, involving in 46% of the cases intron retention events.

The identification of target sites relies entirely on *in silico* prediction, therefore the results need to be taken with some care. However, although these data need further experimental validation, they suggest the presence of this intriguing regulatory mechanism also in grape. Further analyses to validate the miRNA target sites are required to better understand the complexity of miRNA-target interaction and the impact of alternative splicing on modulating miRNA gene regulation.

### Comparison of alternative splicing in different tissues, genotypes and stress conditions

We analysed the expression of the predicted isoforms of each gene across all the samples. For each isoform we estimated the FPKM (Fragment Per Kilo base per Million) expression level using two different programs: Cufflinks and Flux-capacitor (see Methods and Additional file 2: Figure S4 and S5). Both methods gave very similar results; here we refer to those obtained with Cufflinks. We assumed that a FPKM between 1–4 corresponds approximately to 1 RNA molecule per cell [35]. Although we are aware that also low-expressed transcripts may have a functional role, we decided to exclude from our analysis those with a FPKM smaller than 1, because of the uncertainty due to the low number of reads and the approximation of the programs for isoform quantification would yield to low quality results.

**Figure 3:**
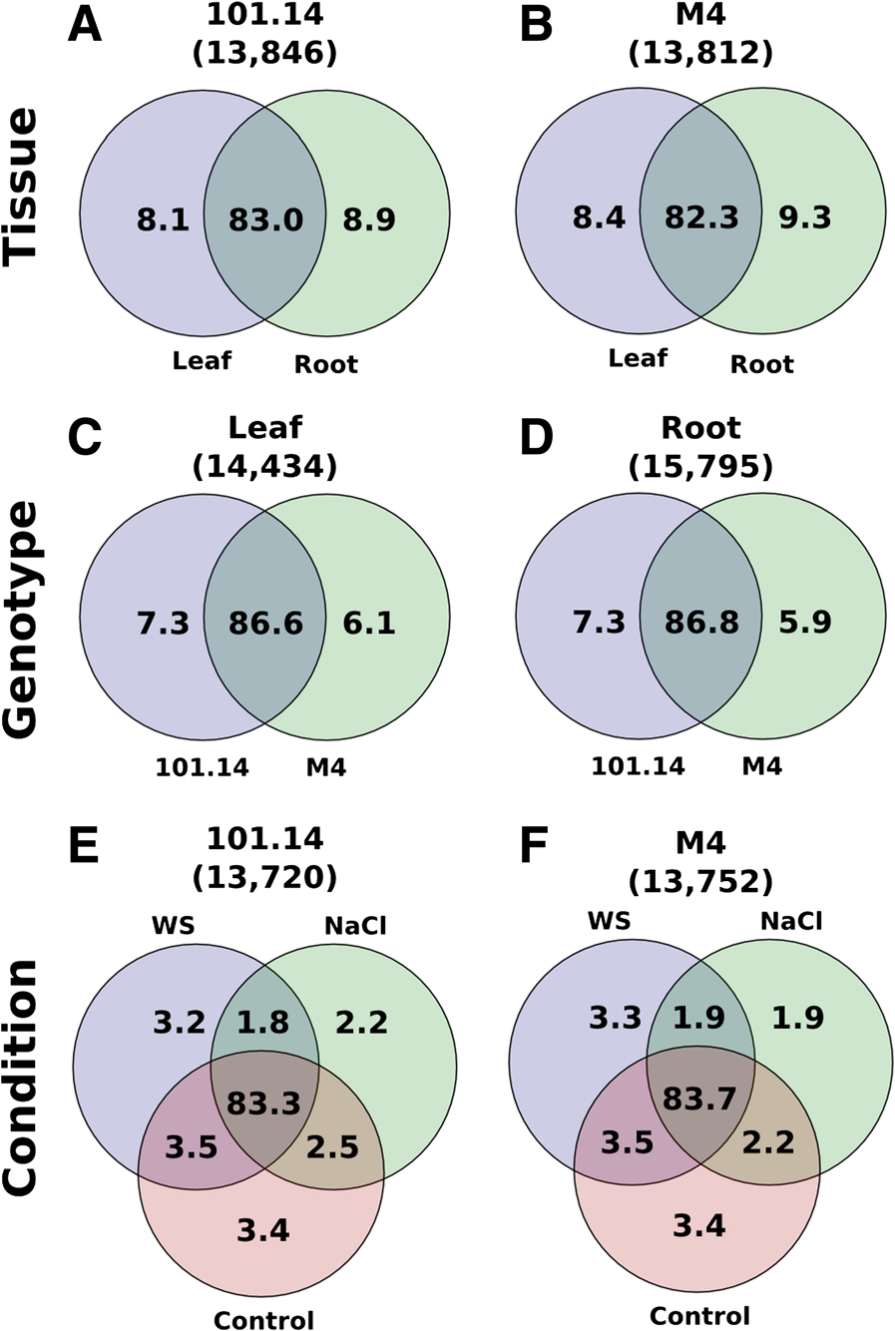
**Isoforms shared between different tissues, genotypes and conditions.** Venn diagrams showing the percentage of different isoforms that are shared comparing different tissues **(A and B)**, genotypes **(C and D)** and environmental conditions **(E and F)**.

The first aspect that we investigated was the number of different isoforms that can be identified comparing different tissues, genotypes and stress conditions. We grouped the samples into three main categories: tissue (leaf and root, Figure 3, panel A and B), genotype (101.14 and M4, Figure 3 panel C and D) and stress conditions (salt-stress, water-stress and controls, Figure 3, panel E and F) and counted how many transcript variants are shared among the different datasets. Cabernet Sauvignon berries were not considered in this analysis because a comparable berry dataset was not available for 101.14 and M4 genotypes. The analysis was performed considering only the genes expressed across all the samples in order to minimize the bias due to the genes that are turned off. In Figure 3 it can be seen that tissues show the highest difference between alternative isoforms, with more than 8% of different variants; genotypes show between 6 to 7% of non-shared variants, while stress conditions show between 4 to 7% (summing up the contribution from control samples). The observation that the extent of change in alternative splicing due to stress is similar to that seen in different tissues is a clear indication of its role in stress response. It should be considered that the status of a transcript being turned on or off depends on its detectability that in turn is dependent on the coverage. As a result we would expect that genes expressed at a low level would produce a minor number of detectable isoforms. Indeed, we found a correlation between the number of identified isoforms and the level of gene expression (Additional file 2: Figure S6).

Genotypes also exhibit considerable variability in splicing. It is interesting to note that the number of different isoforms is greater between genotypes than between plants undergoing different stress conditions. Overall these data indicate that to better understand the molecular bases of phenotypic traits we should also consider the differences in alternative splicing.

**Figure 4:**
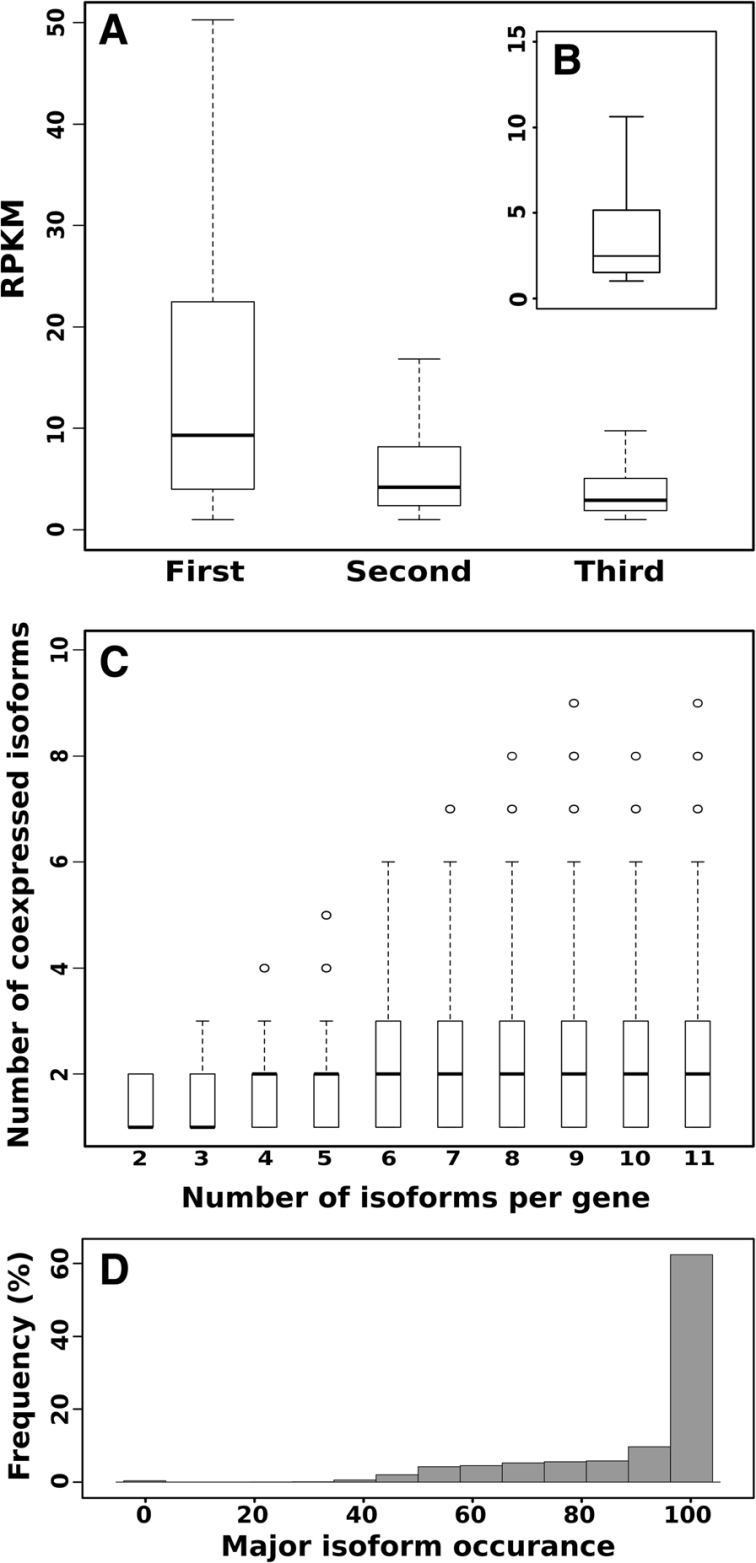
**Isoforms expression analysis. (A)** FPKM value distribution of the first, second and third most abundant isoform within each sample. **(B)** Distribution of the ratio between expression values of the first and second most abundant isoforms. **(C)** Number of co-expressed isoforms compared to the number of isoforms per gene. **(D)** Frequency of the major isoform across the samples.

When we compared the relative abundance of the variants of individual genes, we found that in most cases there is a single transcript that has a considerable higher level of expression rather than a subset of transcripts with similar expression. Figure 4, panel A shows that the FPKM value distribution of the major transcript has a median value of 9, while the second and third variant have a median value of 4 and 2 respectively. When we calculated the ratio between the expression values of the first and second most abundant isoforms, we found that in 60% of the cases the ratio was higher by at least 2-fold and in 25% of the cases 5-fold (Figure 4, panel B). Next, we verified how many isoforms were simultaneously co-expressed in the different samples (Figure 4, panel C). We noticed that genes tend to co-express a low number of isoforms, typically two, and there seems to be no correlation between number of co-expressed isoforms and number of predicted variants. These results are quite different from those observed in human [20] where genes express many isoforms simultaneously and the number of expressed isoforms is correlated to the number of predicted variants. Finally, we verified if there is a tendency of the major isoform to be recurrent across the different samples. For each gene expressed in at least two samples, we identified the major isoform and counted how many times it was the most abundant across the samples. In 4,777 genes (54%) we found that the same major isoform was expressed across all the samples where the gene is expressed (Figure 4, panel D). Similar studies have been recently reported also for human where a survey on 16 different tissues revealed that 35% of the genes tend to express the same major isoform [21]. This result is strongly correlated to the dataset. The extension of the analysis to more samples would probably reduce the number of genes in which the major isoform remains the same.

### Evolutionary adaptation by tuning the alternative splicing machinery

**Figure 5:**
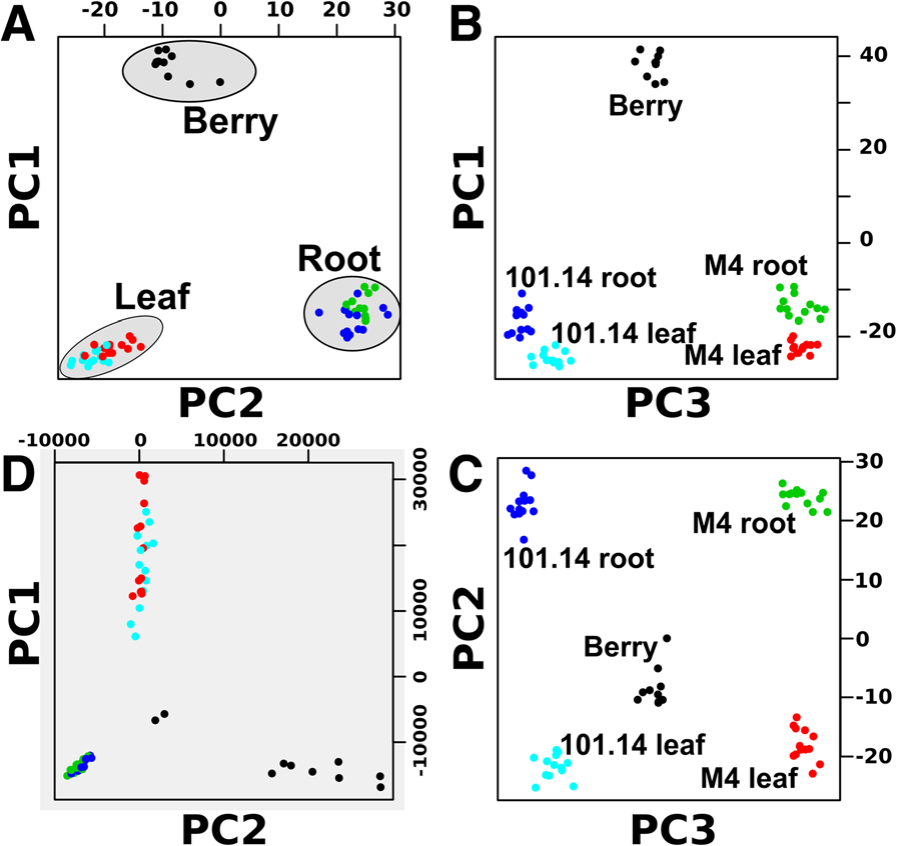
**Isoforms expression principal component analysis. (A,B,C)** Scatter plot of the first three principal component analysis of the expression values ratio between the first two highly expressed isoforms. **(D)** Scatter plot of the first two components of the expression values of the whole gene set. Each dot represents a sample: 101.14 leaf (cyan), 101.14 root (blue), M4 leaf (red), M4 root (green) and Berry (black).

In the previous paragraph we showed that in 54% of the genes the major isoform remains the same across all the samples, while in the remaining 46% the two major isoforms change. To identify possible correlations between alternative splicing and sample types we performed a principal component analysis (PCA). For each gene we considered the two major isoforms amongst all the samples (see Methods). The PCA was performed on the log ratio of the first and the second major isoform expression values. The dots in Figure 5 represent the 62 samples that are visualized according to tissue and genotype (leaf, root and berry tissues and the M4 and 101.14 rootstocks).

The PCA analysis shows some interesting and unexpected results, Figure 5, panel A, B and C show the scatter-plot among the first three PCA components. The first two components (Figure 5, panel A) show that alternative splicing switching events are strongly correlated to the tissue type as the samples from the same tissue tend to cluster together. Unexpectedly, when the third PCA component is taken into account (Figure 5, panel B and C), the samples are further separated according to the rootstock genotype. This suggests the possibility that differences amongst genotypes may also arise from changes in the general splicing regulation program, thus supporting the idea that the evolutionary adaptation to a sudden and strong selective pressure (such as domestication) may be achieved by modifications of the splicing machinery.

To further investigate this hypothesis we considered the serine/arginine-rich proteins (SR), that are known to be involved in pre-mRNA splicing processes and regulate alternative splicing by changing the splice site selection in a concentration dependent-manner. Several studies demonstrated that SR proteins are differentially expressed in different tissues and cell types, and that plant SR genes can produce alternative transcripts with a level of expression that is controlled in a temporal and spatial manner [49].

**Figure 6:**
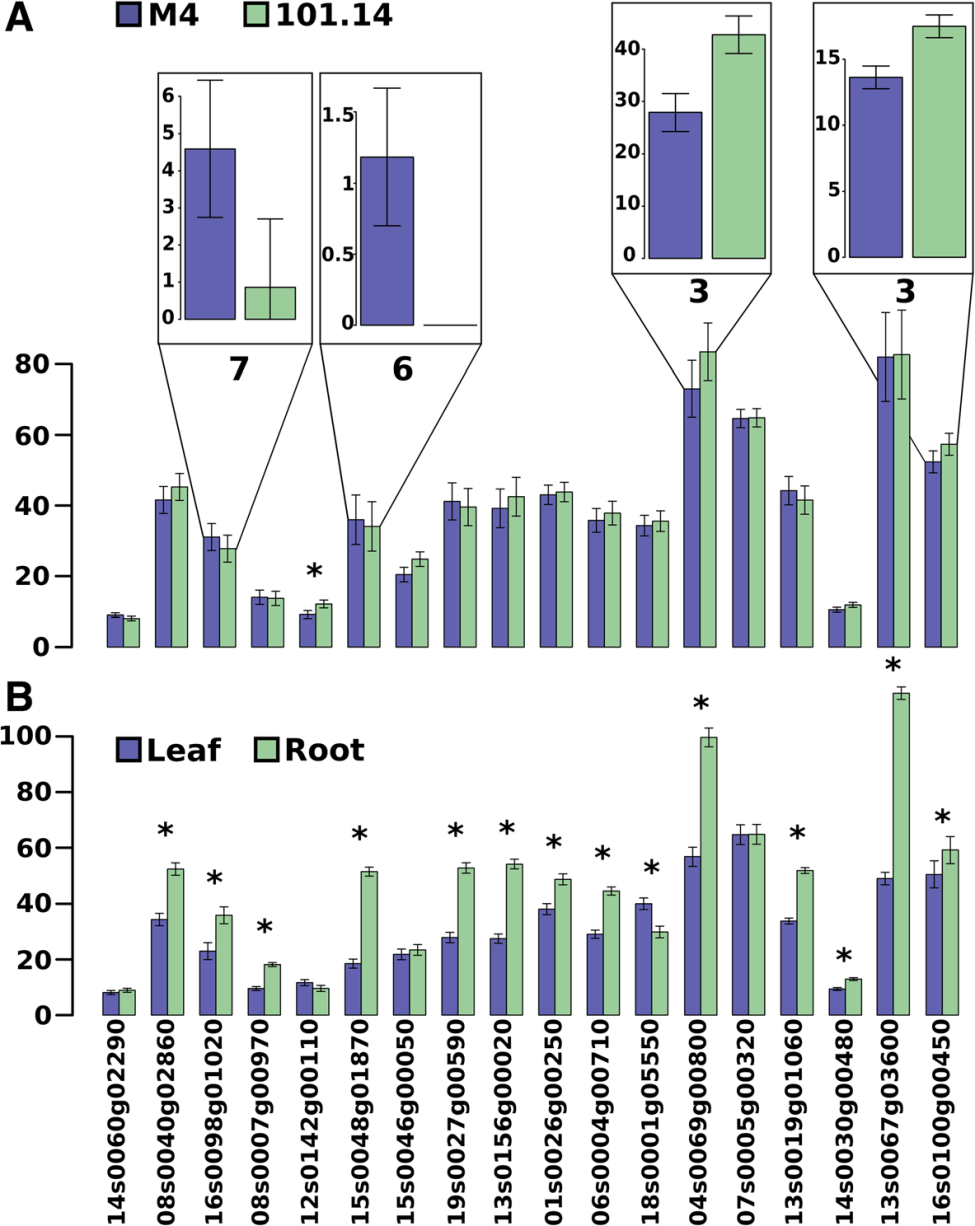
**Differential expression of splicing factor genes in different tissues and genotypes.** Splicing factor average expression value (FPKM) grouping the samples according to genotype **(A)** or tissue **(B)**. Boxes on panel **A** shows the expression levels of the variants that were significantly expressed between genotype. The number above each box represents the number of the isoform. Stars over the bar plots indicate the comparisons that resulted significantly different (t-test with a *p-value* < 0.05 after FDR correction).

Therefore we investigated if there was a significant difference in the expression level of grape SR genes that could be correlated with a different splicing program in tissues and genotypes. Firstly, we performed a blast similarity search (e-value cutoff 1e-^5^) using the 19 SR proteins identified in *Arabidopsis*[50] to identify the orthologous sequences in the grape genome. We were able to identify 18 grape genes as reported in Table 3. Secondly, we compared the mean expression values of the genes grouping the samples according to genotype and tissue type. When the samples are grouped according to genotype, we did not find any significant difference between the genes of the two groups, with the exception of the gene VIT_212s0142g00110 (*p-value* 0.01); however, when the analysis was done taking into consideration the expression level of the isoforms, we found four differentially expressed variants (Figure 6, panel A): VIT_216s0098g01020.7 (*p-value* 0.037), VIT_215s0048g01870.6 (*p-value* 0.003), VIT_204s0069g00800.3 (*p-value* 4.8e^-5^) and VIT_216s0100g00450.3 (*p-value* 0.0007). Finally, when the samples were grouped according to tissue, we found that almost all the SR genes were markedly differentially expressed (15 out 18, Figure 6, panel B), thus confirming the results shown in Figure 5 and indicating that switches in alternative splicing plays a very important role in the definition of tissues and to a lesser extent in genotypes.

**Table 3:** **Grape splicing factors and homologous genes in** ***Arabidopsis***

**Grape**	**Arabidopsis**	**Evalue**	**Gene symbol**	**Description**
VIT_201s0026g00250	AT1G23860	6e-38	SRZ-21	RS-containing zinc finger protein 21
VIT_204s0069g00800	AT4G31580	4e-48	SRZ-22	Serine/arginine-rich 22
VIT_206s0004g00710	AT3G13570	5e-79	SCL30A	SC35-like splicing factor 30A
VIT_207s0005g00320	AT1G02840	2e-75	SR1	RNA-binding (RRM/RBD/RNP motifs) family protein
VIT_208s0007g00970	AT1G16610	8e-10	SR45	Arginine/serine-rich 45
VIT_208s0040g02860	AT2G37340	4e-50	RSZ33	Arginine/serine-rich zinc knuckle-containing protein 33
VIT_212s0142g00110	AT5G64200	2e-54	SC35	Ortholog of human splicing factor SC35
VIT_213s0019g01060	AT3G55460	4e-51	SCL30	SC35-like splicing factor 30
VIT_213s0067g03600	AT2G37340	3e-70	RSZ33	Arginine/serine-rich zinc knuckle-containing protein 33
VIT_213s0156g00020	AT1G55310	1e-13	SR33	SC35-like splicing factor 33
VIT_214s0030g00480	AT5G18810	1e-44	SCL28	SC35-like splicing factor 28
VIT_214s0060g02290	AT1G09140	1e-77	SR30	SERINE-ARGININE PROTEIN 30
VIT_215s0046g00050	AT2G46610	7e-83	RS31a	RNA-binding (RRM/RBD/RNP motifs) family protein
VIT_215s0048g01870	AT5G52040	1e-85	RS41	RNA-binding (RRM/RBD/RNP motifs) family protein
VIT_216s0098g01020	AT3G49430	5e-90	SRp34a	SER/ARG-rich protein 34A
VIT_216s0100g00450	AT5G52040	5e-97	RS41	RNA-binding (RRM/RBD/RNP motifs) family protein
VIT_218s0001g05550	AT5G64200	6e-53	SC35	Ortholog of human splicing factor SC35
VIT_219s0027g00590	AT1G16610	1e-71	SR45	Arginine/serine-rich 45

We also performed a PCA on the expression pattern of genes rather than isoforms and we found that tissues are resolved by the first two components (Figure 5, panel D), while genotypes cannot be resolved, even when the second and third components are considered (Additional file 2: Figure S7). We can conclude that the two different genotypes showed more marked differences in alternative splicing than in change in the level of gene expression.

### Non coding transcripts

To identify long non coding transcripts (lncRNA) we developed a stringent filtering pipeline to discriminate between coding and non coding sequences and to eliminate possible errors of assembly. Briefly, we identified putative lncRNAs based on their expression level and genomic context and only if they had no coding potential, no possible homology with proteins or protein domains and no homology with repeated sequences. LncRNAs can be classified as natural antisense transcripts (NATs), long intronic noncoding RNAs and long intergenic noncoding RNAs (lincRNAs) according to their genome location. Depending on the type of lncRNA, we applied different filters to avoid false positives due to several possible sources of errors, as for example missing UTRs or intron retention events. Further details can be found in the materials and methods section.

**Figure 7:**
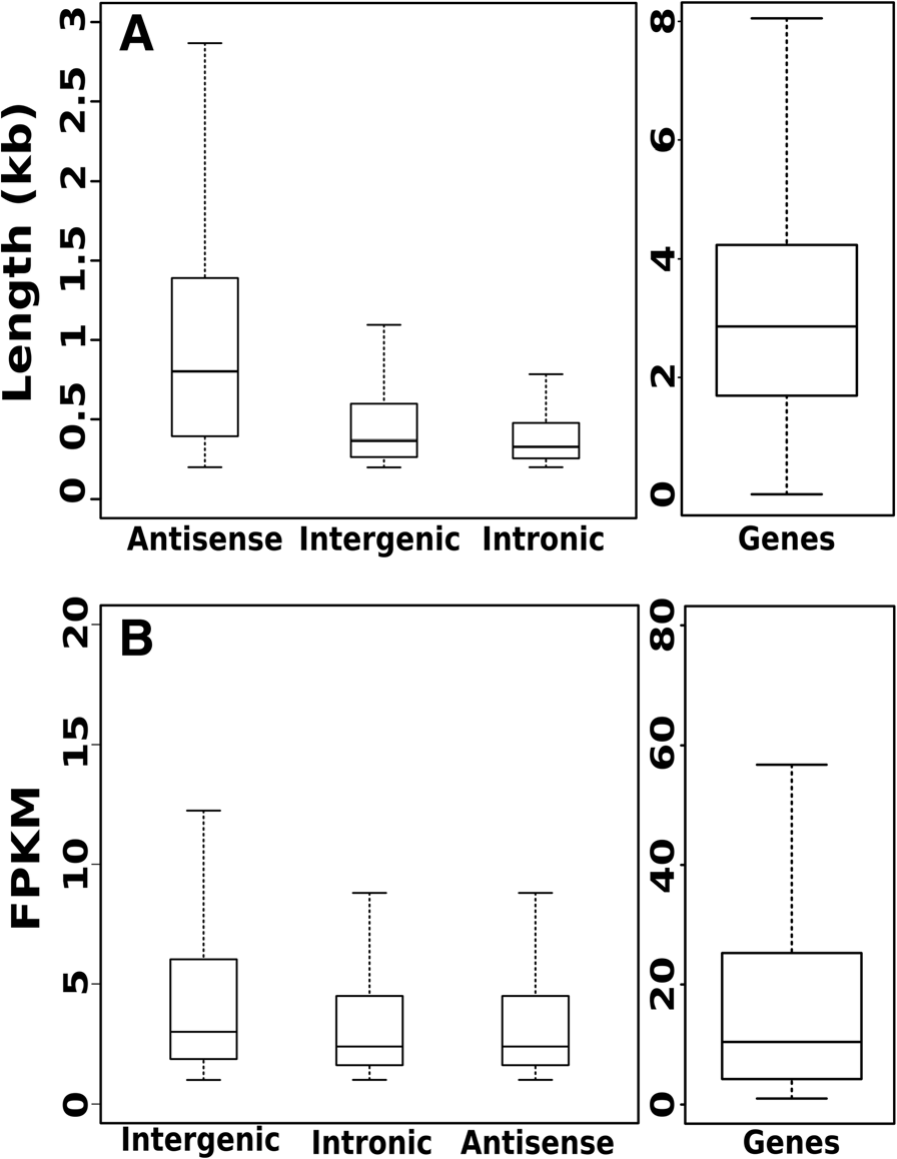
**Long non coding expression and size distribution. (A)** Box-plot of the long non coding size distribution compared to the coding sequence length. **(B)** Box-plot of the long non-coding expression value distribution compared to the coding sequence expression.

This procedure led to the identification of 3,336 long non coding RNA divided into 526 intronic, 1,992 intergenic and 818 antisense transcripts. We analysed the structure, the expression level and the conservation of these lncRNA. We found that grape lncRNA were on average smaller than protein coding genes (mean length of 1,016 nt, 426 and 408 for antisense, intronic and intergenic lncRNAs versus 3,232 nt for protein coding genes). Moreover we found a considerable difference between the length of antisense lncRNA and the other two types of long non coding RNA (Figure 7, panel A). We found that grape lncRNAs are generally monoexonic and that only 11% of intergenic, 1.3% of intronic and 5% of antisense lncRNAs have more than one exon. Consequently to this monoexonic structure, we found that only a low number of lncRNAs undergoing alternative splicing: 40 intergenic lncRNA produced 97 different isoforms while 12 antisense lncRNAs produced 23 variants. We did not detect any isoforms for intronic lncRNAs. These findings are quite different from what was found in human were the majority of lincRNAs are composed by two exons [51].

To verify if the high number of monoexonic lincRNAs was due to low coverage problems, we looked for a possible correlation between lincRNA structure and level of expression. As shown in Additional file 2: Figure S8, the expression level distribution of both monoexonic and multiexonic lincRNAs is quite similar, suggesting that monoexonic lincRNAs structure is not due to low sample coverage.

We performed a similarity comparison between grape lncRNA and the long non coding RNA identified in *Arabidopsis*[13]. Despite the use of a relaxed e-value threshold (blast e-value cutoff lower or equal to 1e^-5^), we identified a very small number of matches. In details we found that only 61 intergenic, 6 intronic and 120 antisense non coding transcripts had at least one match. This result confirms the observation that very few lncRNAs are clearly conserved across species [30,37].

Analysis on the expression level revealed that lncRNAs are on average 10-fold less abundant than protein coding genes (Figure 7, panel B). Similar results have also been found both in *Arabidopsis*[13] and in vertebrates [29,30,37] suggesting that short sizes and low expression levels may be general characteristics of long non-coding RNA and probably related to their differences in biogenesis, processing, stability and function compared to mRNA.

**Figure 8:**
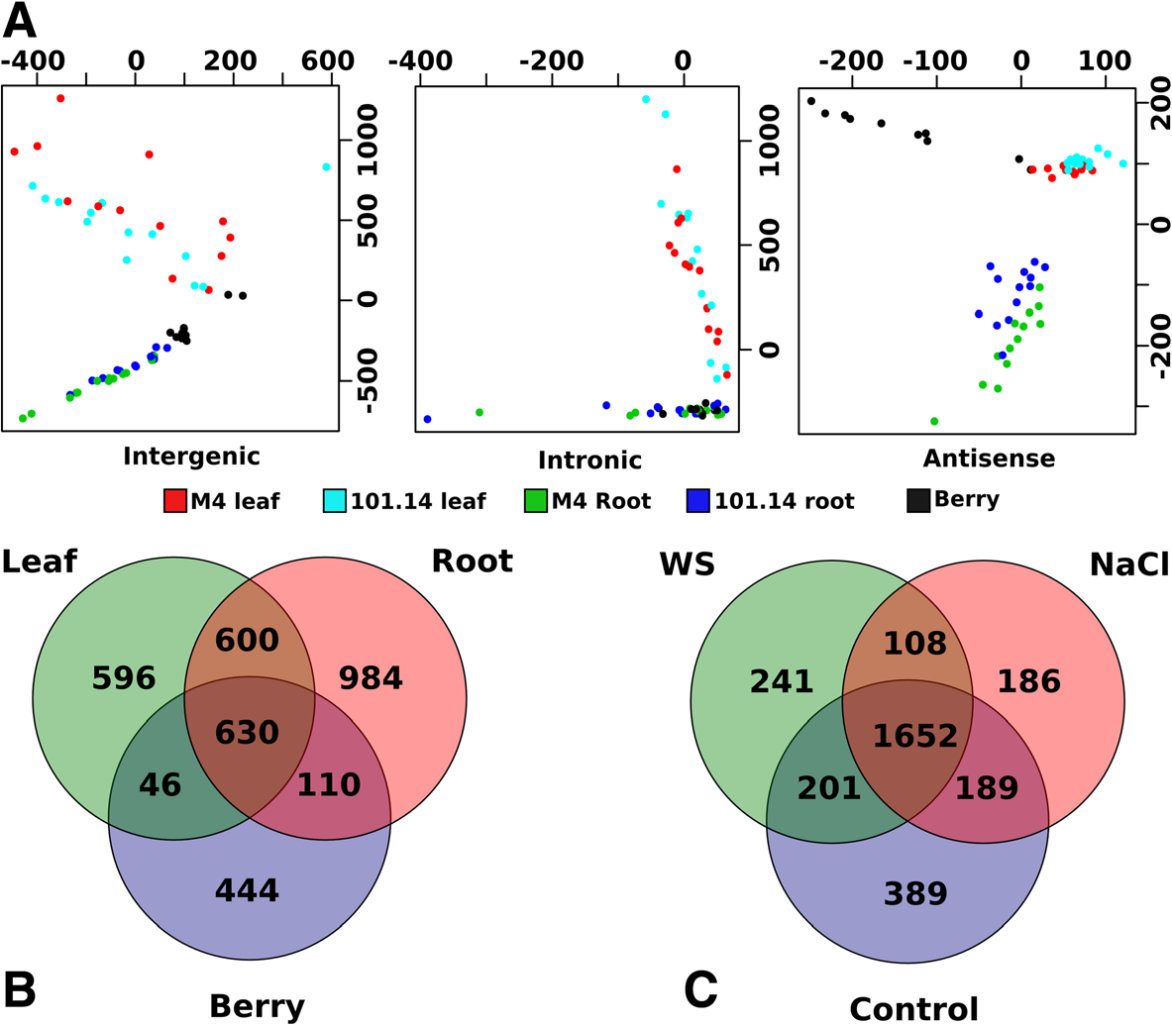
**Long non coding expression analysis. (A)** Principal component analysis of the expression values of the three different categories of lnRNAs. **(B)** Venn diagrams showing the distribution of lnRNA among tissues **(B)** and stressed conditions **(C)**.

We performed a principal component analysis of the expression values of lncRNAs across all the samples. Figure 8, panel A shows that the first two PCA components clearly indicate a tissue-specific expression pattern. Moreover we investigated the expression of lncRNAs in relation to stress conditions. Strong evidence support the hypothesis that long non coding transcripts are involved in the response to different stresses, including biotic stresses and pathogen infections [13,14]. PCA analysis however was unable to efficiently separate the samples according to the stress condition, indicating that the tissue-specificity has a stronger effect on lncRNA expression regulation. Nevertheless Venn diagrams of lncRNAs distribution according to tissue (Figure 8, panel B) and stress conditions (Figure 8, panel C), show that even though many lncRNAs are tissue-specific, as already suggested by PCA analysis, there is a considerable number of lncRNA that are induced by stress conditions. We found 241 lncRNAs that are uniquely induced during water stress, 186 during salt stress and 108 that are common between the two stress conditions.

### Data availability

The new v2 gene prediction, together with the long non coding sequences and other useful information resources on *Vitis vinifera* are available for download as flat files in popular file formats (gff3, fasta) at http://genomes.cribi.unipd.it/grape. Data are also accessible through a web based informatics infrastructure that integrates the data giving the possibility to visualize and further analyze the available data. The web resource hosts a genome browser, a blast server to perform similarity search against the genome, genes and long non coding sequences, and an advanced query platform to perform complex queries.

The RNAseq data used in this study has been deposited at the NCBI Short Read Archive (http://www.ncbi.nlm.nih.gov/Traces/sra/sra.cgi) under the following SRA accession: SRA110531 and SRA110619.

## Conclusions

In this paper we present an improved grape gene prediction, named v2, based on the incorporation of a great amount of RNAseq data. A considerable number of new genes have been identified, including many genes related to lncRNAs. The sequencing libraries were produced with a procedure that assured a high directional accuracy, that is particularly important in the annotation of lncRNA and for the identification of anti-sense RNAs. Furthermore, with this study we have produced, for the first time in grape, a comprehensive description of alternative splicing in different tissues, genotypes and stress conditions. As observed in *Arabidopsis* and now in grape, alternative splicing and non coding RNAs contribute significantly to the transcriptional complexity and they should be taken into consideration in genome wide transcriptomic studies.

In plants, in particular in *Arabidopsis*, it has been shown that many genes undergo regulated alternative splicing. Serine/arginine-rich (SR) and heterogeneous nuclear RNP (hnRNP) proteins are the main splicing factors (SF) involved in constitutive as well as regulated AS. The level of several SFs changes in different plant tissues [52] and in this study (Figure 7, panel B) we showed that this is true also in grape. Similarly, stress and environmental conditions produce specific SF changes [53,54]. Thus, changes in the SF profile, driven by developmental and environmental conditions, contribute to the definition of the splicing specifications to be applied in different circumstances (for a review see [55]).

Differences in alternative splicing have also been described between different ecotypes of *Arabidopsis*[56], giving support to the argument that changes in splicing may contribute to the evolutionary adaptation process. A question that is still open is whether the changes in splicing observed in different *Arabidopsis* ecotypes are due to specific alterations of the splicing sites or if they could be determined also by general modifications of the splicing machinery.

In this paper we show that like *Arabidopsis* ecotypes, also grape genotypes exhibit some splicing specificity. Interestingly, when we investigated if this could be due to changes in the splicing machinery we saw only minor differences in the level of expression of the 18 SR genes of grape (Figure 7, panel A), but when we investigated at the level of individual SR isoforms we found some very striking changes (Figure 7A, top frames). Another interesting observation is that the SF isoforms that are differentially expressed in the two genotypes did not show differential regulation in tissues. This suggests a possible mechanism of perturbation of the splicing machinery that would interfere only marginally with the splicing specifications required in different tissues. A more detailed description of these differences is given in Additional file 2: Table S5 and S6 and Figure S9-S12.

The finding that plants that practically belong to the same biological species have different splicing machineries is very intriguing and leads to interesting considerations and hypotheses. Since SFs affect also the splicing of their own transcripts, small changes of the splicing machinery may reshape the AS profiles to new points of equilibrium where each SF panel will produce the same SF isoforms. A more detailed knowledge of the specific role of each SF will help to better understand this problem.

Finally, it should be considered that small changes of the splicing machinery could play an important role in evolutionary adaptation, providing an easy and quick generation of several “variations on the theme”, using parts that have already been tested (the isoforms), but changing their assortment. This could be particularly relevant in the response to sudden and strong selective pressures. Therefore, it would be interesting to verify whether some of the intraspecific AS differences that are often reported in different plant species and cultivars are due to changes of individual splicing sites or if they could result from the tuning of the splicing machinery.

## Methods

### Dataset

For a detailed description of the experimental design see [38]. Briefly, 108 plants of 101.14 and M4 were grown in a greenhouse and divided into 6 pools and exposed to drought and salt stresses. The water stress treatment was imposed decreasing gradually in 10 days the water quantity from 80% to 30% of the field capacity. Leaves and roots tissues were collected at 2 (time point T1), 4 (T2), 7 (T3) and 10 (T4) days after the beginning of the stress experiment.

Salt stress treatment was imposed adding daily 5 mmol of NaCl to plants with a water availability equal to 80% of the field capacity. Leaves and roots tissues were collected from plants at 2 (T1), 4 (T2) and 10 (T3) days after the beginning of the stress experiment. At each time point of the two experiments, mRNA from non-stressed plants of both the two rootstocks grown with a water availability equal to 80% of the field capacity were sampled as controls.

All samplings were performed in two biological replicates producing a total of 52 leaves and 52 root samples. Total RNA of all samples was extracted from leafs and roots using the “Spectrum^TM^ Plant total RNA Kit” (Sigma) according to manufacturer instructions. Moreover we included 20 RNAseq berry samples from *Vitis vinifera* L. cv Cabernet Sauvignon (CS) grafted onto 1103P and M4 rootstocks (Pasqua vigneti e cantine, Novaglie VR, Italy). Grapevines were grown in well-watered conditions. Whole berries were collected from 1103P (*V. berlandieri x V. Rupestris)*, and M4 bunches at 45, 59, 65 days after full bloom (DAFB), in correspondence to the end of lag phase when most of grape berries reached veraison. The other samples (separating skin and pulp) were collected at 72, 86 and 100 DAFB. Total RNA was extracted from the above cited samples using the method described in [57]. These samples were sequenced using the same technology.

### Library preparation and sequencing

The mRNA was extracted from total RNA using the Dynabead mRNA Direct kit (Invitrogen pn 610.12). We obtained a variable quantity of mRNA from the total RNA that ranged from 0.4 to 1.6%, with a mean value of 0.8%. We prepared the samples for Ligation Sequencing according to the SOLiD Whole transcriptome library preparation protocol (pn 4452437 Rev.B). The samples were purified before RNAse III digestion with Purelink RNA micro kit columns (Invitrogen, pn 12183–016), digested from 3 min to 10 min according the starting amount of mRNA, retrotranscribed, size selected using Agencourt AMPure XP beads (Beckman Coulter pn A63881) and barcoded during the final amplification. The libraries were sequenced using Applied Biosystems, SOLiD™ 5500XL, which produced stranded paired end reads of 75 and 35 nucleotides for the forward and reverse sequences respectively. The average insert size was 114 with a standard deviation of 49 bp, as calculated on the aligned paired ends.

### RNAseq genome alignment and filtering

The reads were aligned to the reference 12X grape genome using PASS aligner [40]. The percentage identity was set to 90%, and one gap was allowed. For each read we considered only the best alignment. In the case of reads mapping in multiple sites, for gene prediction we considered them all, while for gene expression we considered only the reads mapping to unique genomic positions. The spliced reads were identified using the procedure described in PASS manual (http://pass.cribi.unipd.it). We applied different filters to spliced reads mapping in order to reduce the number of false positive. These include the rejection of reads with an overlap length less than 15 bases on one exon and with a total alignment length of less than 40 bases. Additional filters based on read coverage to improve the accuracy of the potential new introns: 1) the splicing site need to be confirmed by reads from both the replicates with a total read coverage of at least 4; 2) in case the reads come from only one replicate, the read coverage was increased to 10.

### Transcript reconstruction and gene prediction update

To improve read coverage, the biological replicates were merged together. For each RNAseq library, we reconstructed the transcripts using three different programs: Cufflinks version 2.0.2 [36], Isolasso [41] and Scripture [37]. The programs were run using default parameters except for Cufflinks option “-j” that was set to 0.3 in order to increase the minimum depth of coverage in the intronic region and IsoLasso option “-c” that was increased to 5 to adjust the minimum number of clustered reads.

All the transcript models generated by the three programs were compared with each other using Cuffcompare program from Cufflink package. We considered good transcripts only those that were predicted with the same genomic structure by at least two different programs. Finally, the transcripts that were shorter than 150 nucleotides were considered false positives and removed from further analysis.

The obtained dataset was used to update the grape v1 gene prediction. The transcripts were aligned on the genome using PASA [42], an eukaryotic genome annotation tool that uses spliced alignments of transcript sequences to automatically model and update gene structures. PASA updated both the UTR regions and the gene models and identified the alternatively spliced models.

### Identification of new genes

Beside the v1 gene prediction update, we also performed a new gene prediction in order to identify potentially new genes. We used several tools and approaches based both on *de novo* predictor and protein/transcripts sequenced based alignment methods. We used a collection of 1.158.221 non redundant *vidiriplantaea* proteins and 7.897.336 *eudicotyledons* ESTs downloaded from NCBI ftp site. The sequences were aligned on the genome using initially a blast program [58] to quickly identify the similarity regions and Exonerate [59] software to refine the alignment. We used a blast e-value cutoff of 1e^-30^ and 1e^-5^ for protein and nucleotide alignment respectively.

We used Augustus [60]*de novo* gene predictor trained with the assemblies generated by PASA. The training was performed using the automatic procedure available at the Augustus web server [61]. Augustus prediction was performed feeding the program with the whole RNAseq data alignment.

The final gene prediction was obtained using EvidenceModeler (EVM) [62] software in order to combine *ab intio* gene predictions, protein ESTs and PASA assemblies alignments into weighted consensus gene structures. We compared the gene models generated by EVM with the updated grape gene prediction and identified a set of 6,381 new potential genes. We applied several criteria to discriminate between true coding protein genes and non-coding or gene model artefacts. At first, we excluded all the genes showing a similarity with a miRNA gene or predicted to be non-coding by CPC software [63]. Moreover, we performed a further filtering selecting all the genes that fulfil at least one of the following criteria: i) an homologous sequences on another species excluded *V. vinifera* ii) presence of an interproscan predicted domain iii) a RNAseq coverage of at least 50% of the gene model. The final gene set was processed by PASA to add the UTR regions and to calculate the alternatively spliced models.

### Gene annotation

Gene annotation was performed using a combination of software, tools and databases. At first we used InterProScan v4.8 [64], an integrated annotation system that looks for protein domains and functional sites integrating 11 different databases. Secondly we performed a similarity search at the protein level on the grape genes against the non redundant databases using the blast algorithm [58]. Finally we performed gene ontology and KEGG annotation using a local version of blast2go [65].

### Evaluation of the gene prediction quality

To identify fusion/splitting events, we compared the two gene predictions using the cuffcompare program and selected the genes in one prediction overlapping to two or more genes in the other gene dataset. We used the BLASP program to search each group of fused/split genes against the *Arabidopsis* proteome using a evalue cut-off equal or lower than 1e-5. If a v2 merged gene showed the same best hit than the v1 split genes, we considered it as correct. On the other hand if the split v2 genes showed two different best hits we considered them better than the v1 merged gene. When one of the split genes do not show any similarity with Arabidopsis proteins, we classified it as “Ambiguous”.

The genes from the two gene predictions mapping on the same genomic locus but with a different coding sequence were evaluated using a method similar to the one described in [66] that implies a 2-steps analysis: at first for each pair of sequences we performed a similarity search against the *Arabidopsis* proteome and we identified the two best hits. If the best hits were the same for both the gene prediction, we re-aligned the two gene models on the reference using a global alignment based on Needleman–Wunsch algorithm implemented in the program *stretcher* from EMBOSS package [67]. Alignments having a percent identity below 30% were removed. In the case of v2 alternative spliced genes, we aligned all the isoforms against the *Arabidopsis* homologous protein and we selected the variant with the highest score. The results of this analysis are reported in Additional file 2: Table S4.

### Isoform quantification and analysis

The transcripts were quantified with two different programs, Cufflinks [36] and Flux-capacitor [68] and all the further analyses were done independently using the results from both the programs. In this manuscript we report the results obtained using Cufflinks. All the other results are available as Additional file 2. The transcript abundances were quantified using both the replicates for each sample. To constrain quantification errors, we considered expressed only those genes with a FPKM value of at least 1 on both the replicates. For the genes that passed this first filter the expression value was calculated as the mean of the FPKM values of the biological replicates.

Transcript switching analysis was performed identifying for each alternative spliced gene those having two major isoforms expressed in the highest number of samples. A major isoform is defined as the transcript with the highest expression value within a given sample. For each gene we calculated the log ratio of the expression values between the two transcripts across all the samples. The obtained dataset was used to perform a principal component analysis using “prcom” function in R environment.

### Splicing factor gene analysis

To assess differences in the expression level of the splicing factor genes between both genotypes and tissues we applied the t-test implemented in the statistical package R. We grouped the samples according to genotype or tissue and we considered as significant only those that had a *p-value* equal or lower than 0.05 after FDR (False Discovery Rate) correction.

### Long non coding prediction

The assemblies generated by Cufflinks were compared to the v2 gene prediction using cuffcompare program. We selected all the sequences with size of at least 200 bp and labelled as intergenic (“u” code), intronic (“i” code) and exonic overlap on the opposite strand (“x” code). In order to discriminate between coding and non-coding sequences we applied the following procedure. First we ran the Coding Potential Calculator (CPC) program [63] to assess the protein-coding potential of the transcripts. The sequences predicted as non-coding were further filtered according to InterProScan annotation [64] and all the sequences with an annotated domain were removed. Next, we checked for the presence of repeated sequences using RepeatMasker program (http://www.repeatmasker.org/). Then, depending on the type of the sequences, we applied some additional filters based on the genomic context. We removed all intergenic transcripts that were located within a 1 kb from the nearest genes. This filter was used to reduce the risk of considering missing UTRs as non-coding transcripts. Moreover, we deleted all the intronic transcripts nearer than 100 base distance from the flanking exons and with a length higher than 30% of the length of the hosting intron. We applied these filters to discriminate between genuine non-coding transcripts and transcripts that arise because of possible non-mature transcripts or intron retention events. Finally to reduce the risk of artefacts, we removed all the antisense transcripts with an overlap shorter that 30% within an exon. We quantified the transcripts abundance with Cufflinks using the biological replicates of each sample. Only the transcripts with an FPKM expression value of at least 1 on both the replicates were considered for further analysis.

## Abbreviations

AS: Alternative splicing; FPKM: Fragment per kilo base per million; PCA: Principal component analysis; DAFB: Days after full bloom; FDR: False discovery rate; CPC: Coding potential calculator.

## Competing interests

The authors declare that they have no competing interests.

## Authors’ contributions

NV performed the bioinformatic analysis and drafted the manuscript. CF and AT set up the gbrowser and the web server. ECC contributed in the drafting of the manuscript and participated in interpreting bioinformatic results. DC contributed to the reads alignment procedure. CB and ML designed and supervised the stress experiments. MC and AV prepared and provided the RNA samples. MD and RZ performed SOLiD sequencing. GV supervised and coordinated the study and drafted the manuscript. All authors contributed in the discussion and interpreting of results and carefully read and approved the final manuscript.

## Supplementary Material

Additional file 1 Excel, new gene set, tabular file with the new identified genes. Click here for file

Additional file 2 Supplementary data. Click here for file

Additional file 3 Excel, overlap between V1 and V2 gene prediction. Click here for file

Additional file 4 Excel, miRNA target site prediction. Click here for file
