# Supplementary material for: A deep survey of alternative splicing in grape reveals changes in the splicing machinery related to tissue, stress condition and genotype
Source: BMC Plant Biol. 2014 Apr 17;14:99. doi: 10.1186/1471-2229-14-99 (PMC4108029; doi:10.1186/1471-2229-14-99)
Supplement: Additional file 2 — Supplementary data. [file 1471-2229-14-99-S2.pdf]

**A deep survey of alternative splicing in grape reveals changes in the splicing machinery related to tissue, stress condition and genotype**

Nicola Vitulo[1], Claudio Forcato[1], Elisa Corteggiani Carpinelli[1], Andrea Telatin[1], Davide Campagna[4], Michela D'Angelo[1], Rosanna Zimbello[1], Massimiliano Corso[2], Alessandro Vannozzi[2], Claudio Bonghi[2], Margherita Lucchin[2,3], Giorgio Valle[1,4,]

1 CRIBI Biotechnology Centre, University of Padua, via G. Colombo 3, 35121, Padova.

2 Department of Agronomy, Food, Natural resources, Animals and Environment, DAFNAE, University of Padua, Agripolis, viale dell'Università 16, 35020, Legnaro, Padova, Italy.

3 CIRVE, Centre for Research in Viticulture and Enology, University of Padua, Agripolis, viale dell'Università 16, 35020, Legnaro, Padova, Italy

4 Department of Biology, University of Padua, via G. Colombo 3, 35121, Padova.

# Supplementary Data

Supplementary Figure S1: GO enrichment....2
Supplementary Figure S2: Analysis of the coding-protein genes with a different structure....3
Supplementary Figure S3: InterProScan annotation comparison between v1 and v2 gene prediciton4
Supplementary Figure S4. Flux-capacitor quantification results....5
Supplementary Figure S5. Flux-capacitor PCA....6
Supplementary Figure S6: Correlation between the number of identified isoforms and gene expression level....7
Supplementary Figure S7: PCA on the expression pattern of genes rather than isoforms. ....8
Supplementary Figure S8: Correlation between lincRNAs structure and expression level....9
Splicing factor isoforms and RRM (RNA Recognition Motif) domain....10
Supplementary Figure S9: VIT_215s0048g01870....10
Supplementary Figure S10: VIT_2160100g00450....10
Supplementary Figure S11: VIT_204s0069g00800....11
Supplementary Figure S12: VIT_2160098g01020....11
Supplementary Table S1: Introns support analysis. ....12
Supplementary Table S2: Types and number of evidence confirming introns....12
Supplementary Table S3: Fusion/splitting events analysis....13
Supplementary Table S4: Analysis of the coding-protein genes with a different structure....13
Splicing factor T-test result tables....14
Supplementary Table S5: analysis performed considering the gene expression level....14
Supplementary Table S6: analysis performed considering the transcript expression level....15

**Supplementary Figure S1: GO enrichment**

Enrichment GO (Gene Ontology) analysis of the new predicted genes.

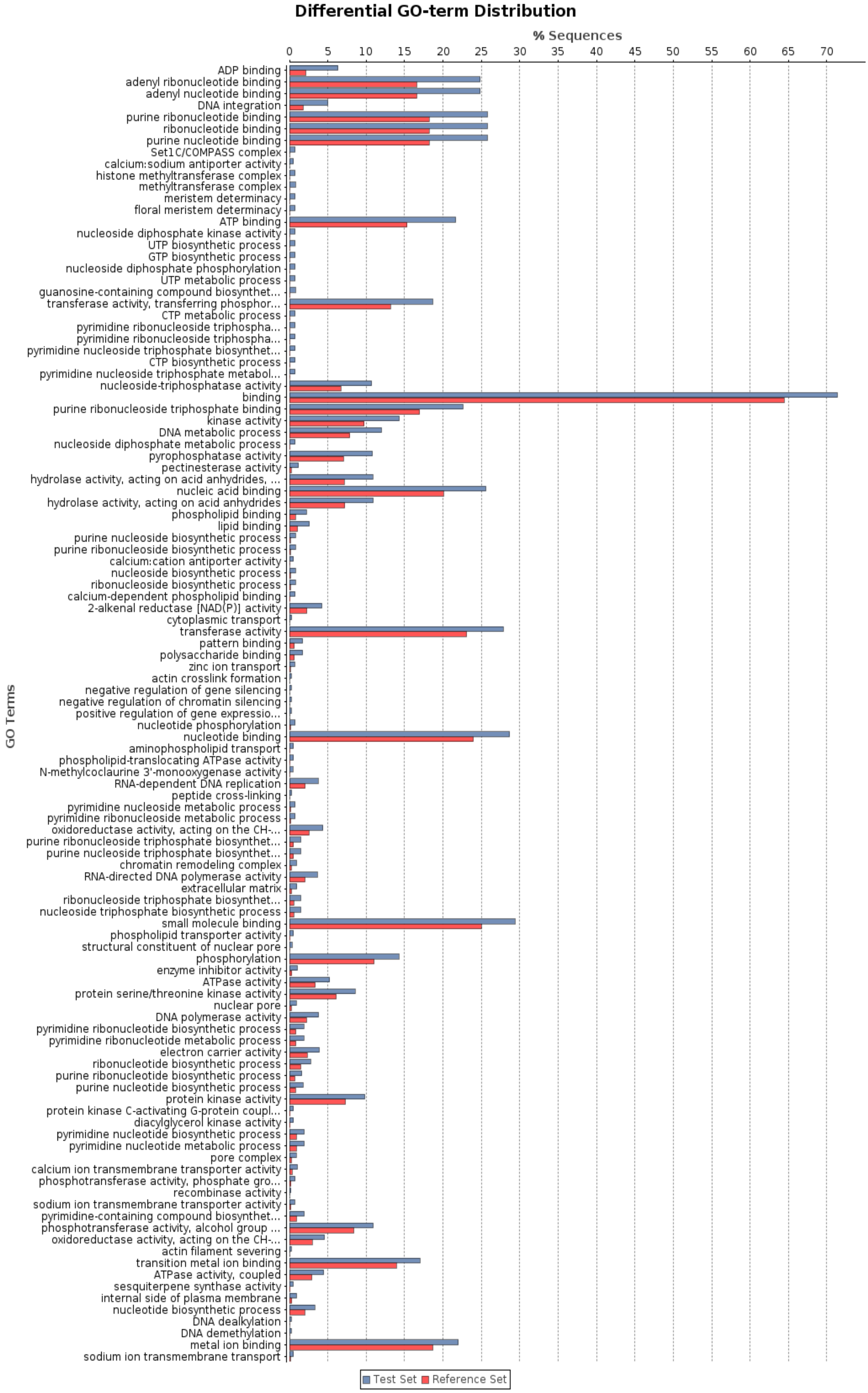

**Supplementary Figure S2: Analysis of the coding-protein genes with a different structure.**

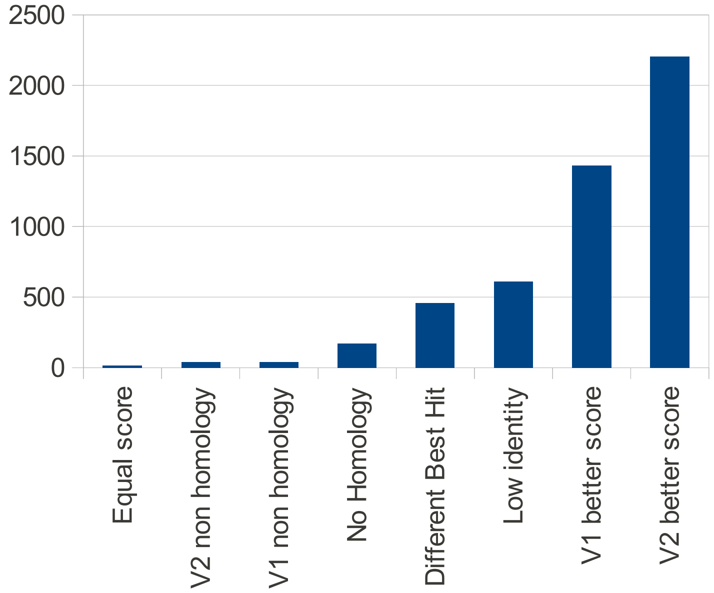

**Supplementary Figure S3: InterProScan annotation comparison between v1 and v2 gene prediciton**

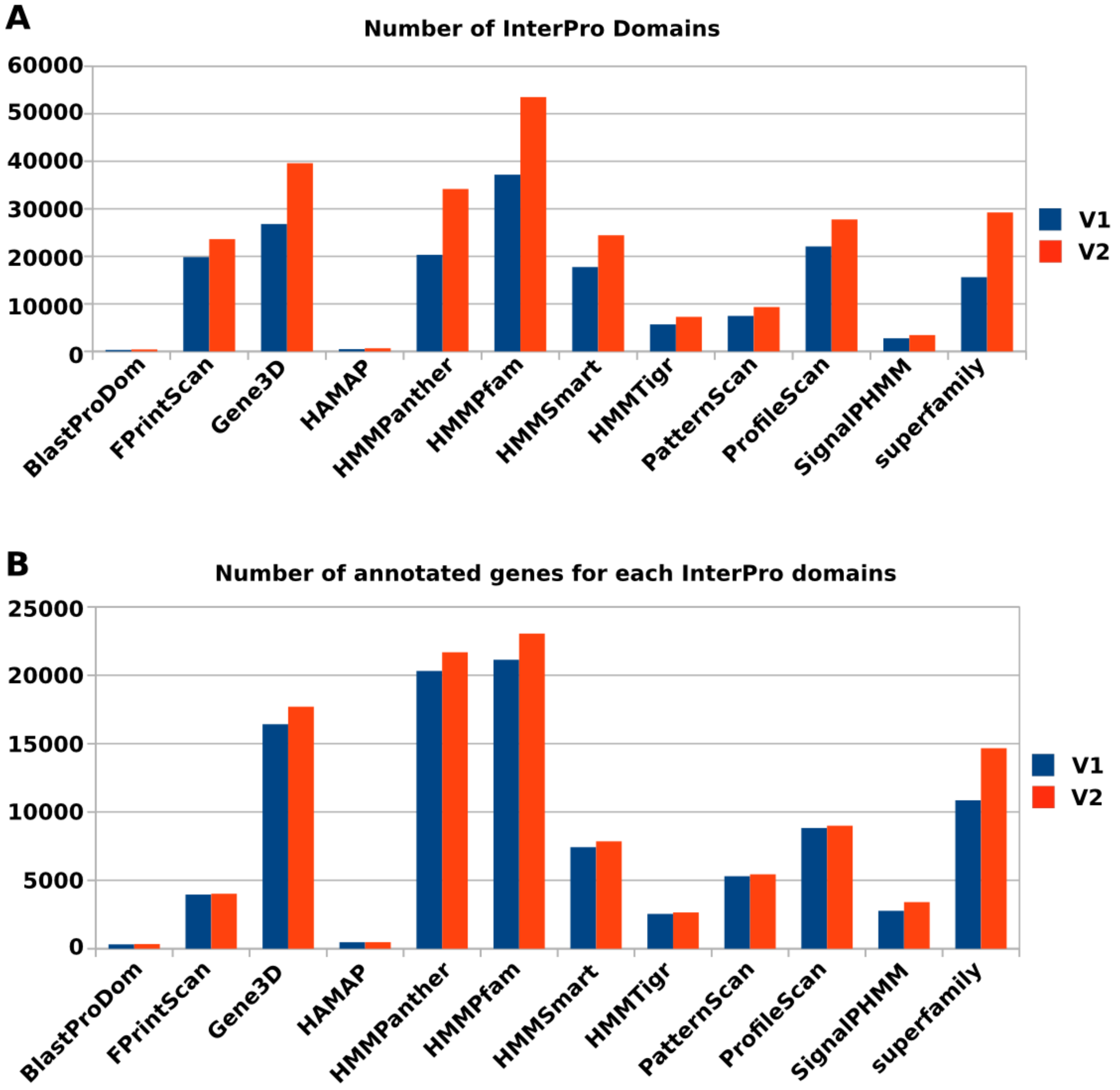

**Supplementary Figure S4. Flux-capacitor quantification results**

(A) RPKM values distribution of the first, second and third most abundant isoform within each samples. (B) Number of co-expressed isoform compared to the number of isoform per gene. (C) Frequency of the major isoform across the samples.

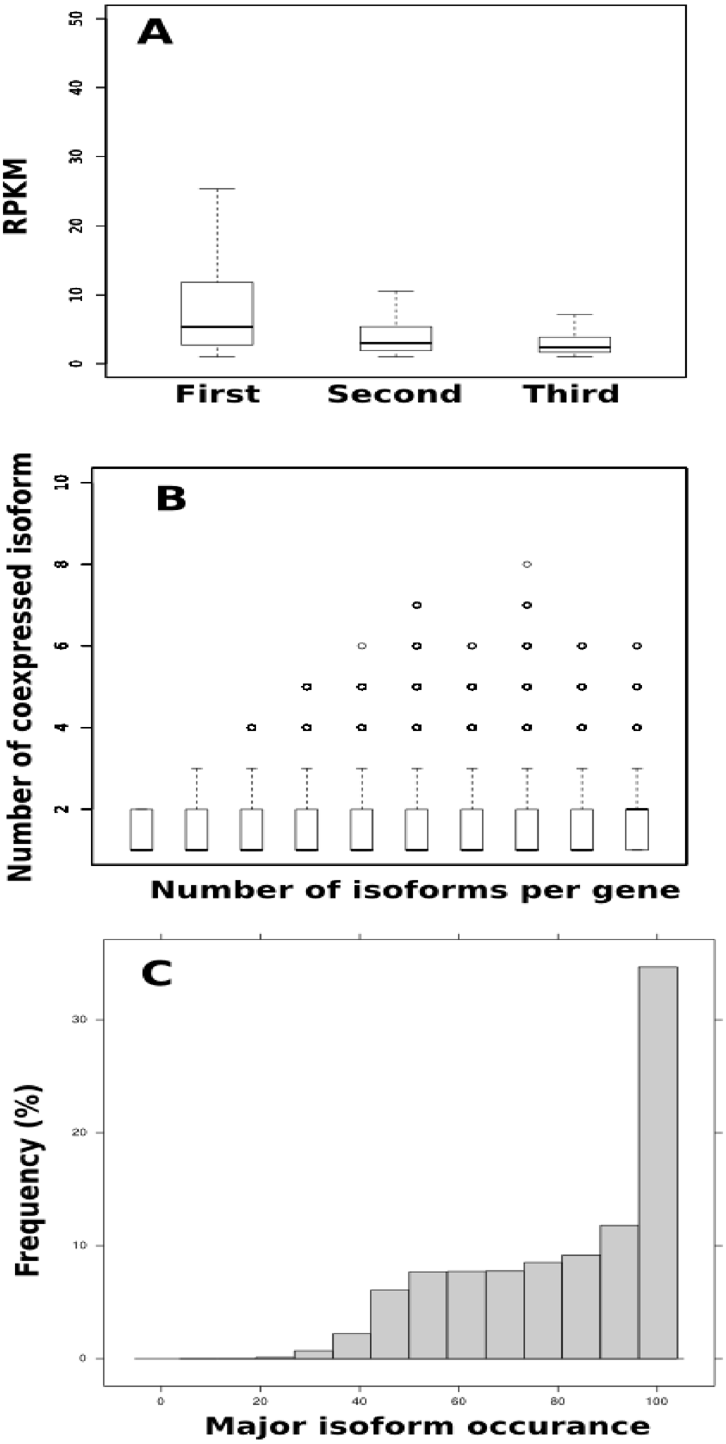

**Supplementary Figure S5. Flux-capacitor PCA**

Scatter plot of the first three principal component analysis of the expression values ratio between the first two highly expressed isoforms.

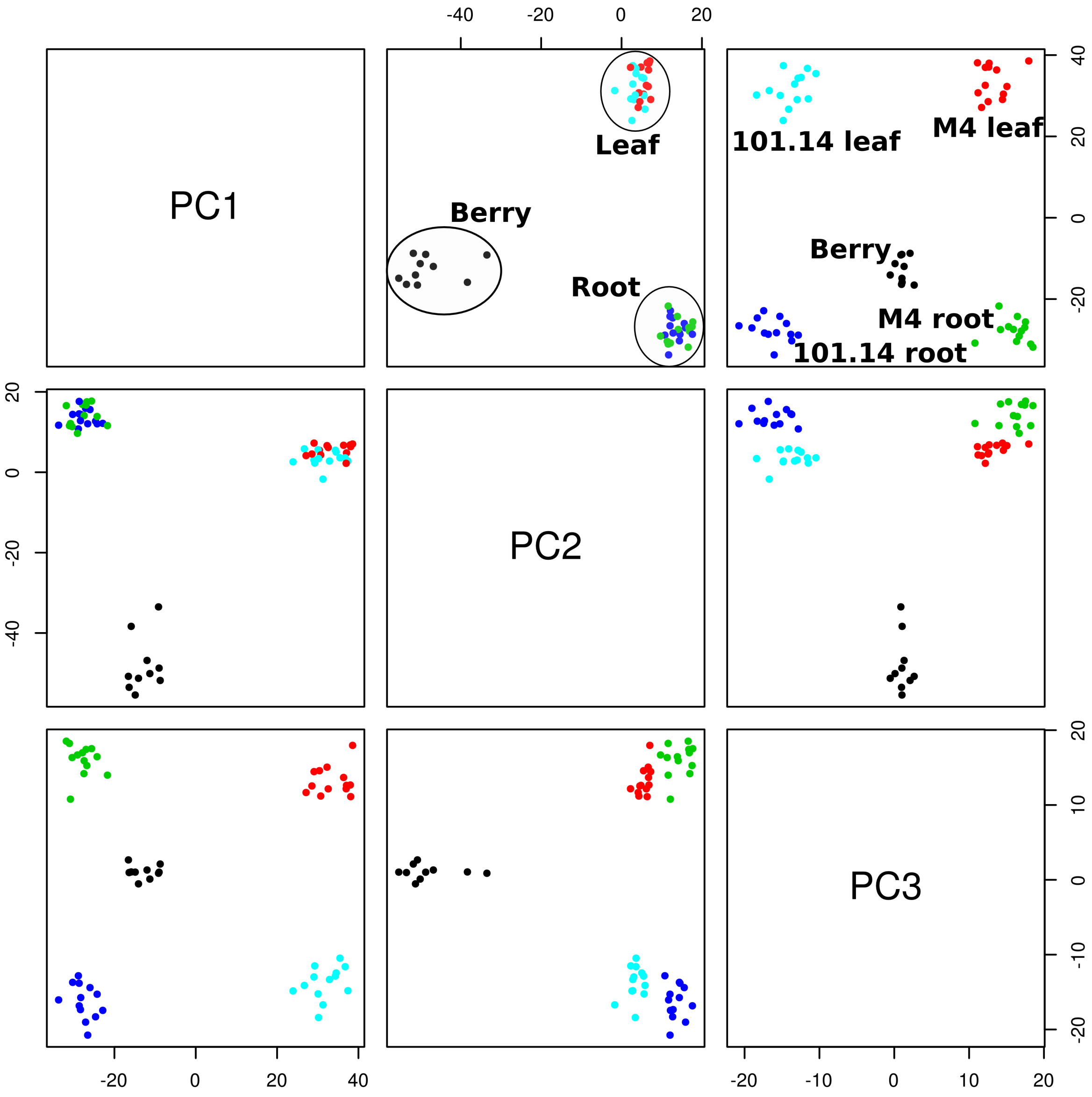

**Supplementary Figure S6: Correlation between the number of identified isoforms and gene expression level**

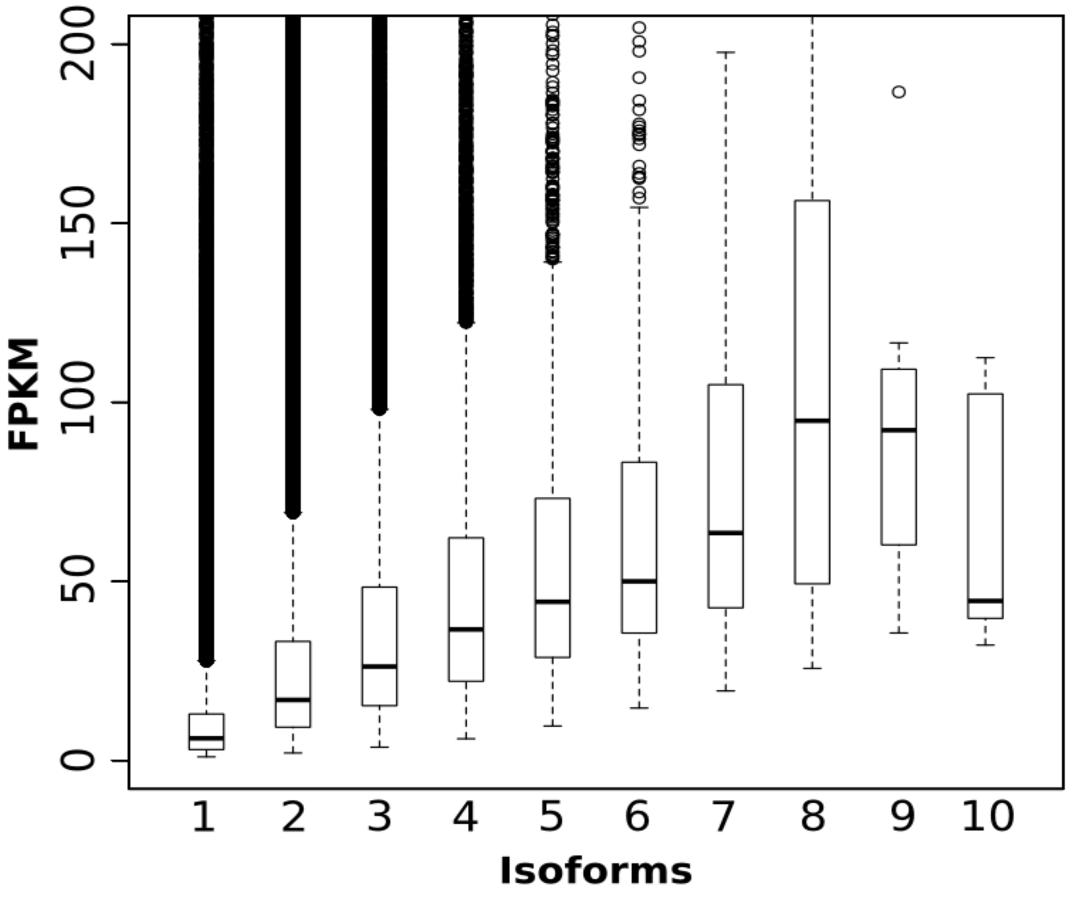

**Supplementary Figure S7: PCA on the expression pattern of genes rather than isoforms.**

Tissues are resolved by the first two components, while genotyoes cannot be resolved, even when the second and third components are considered

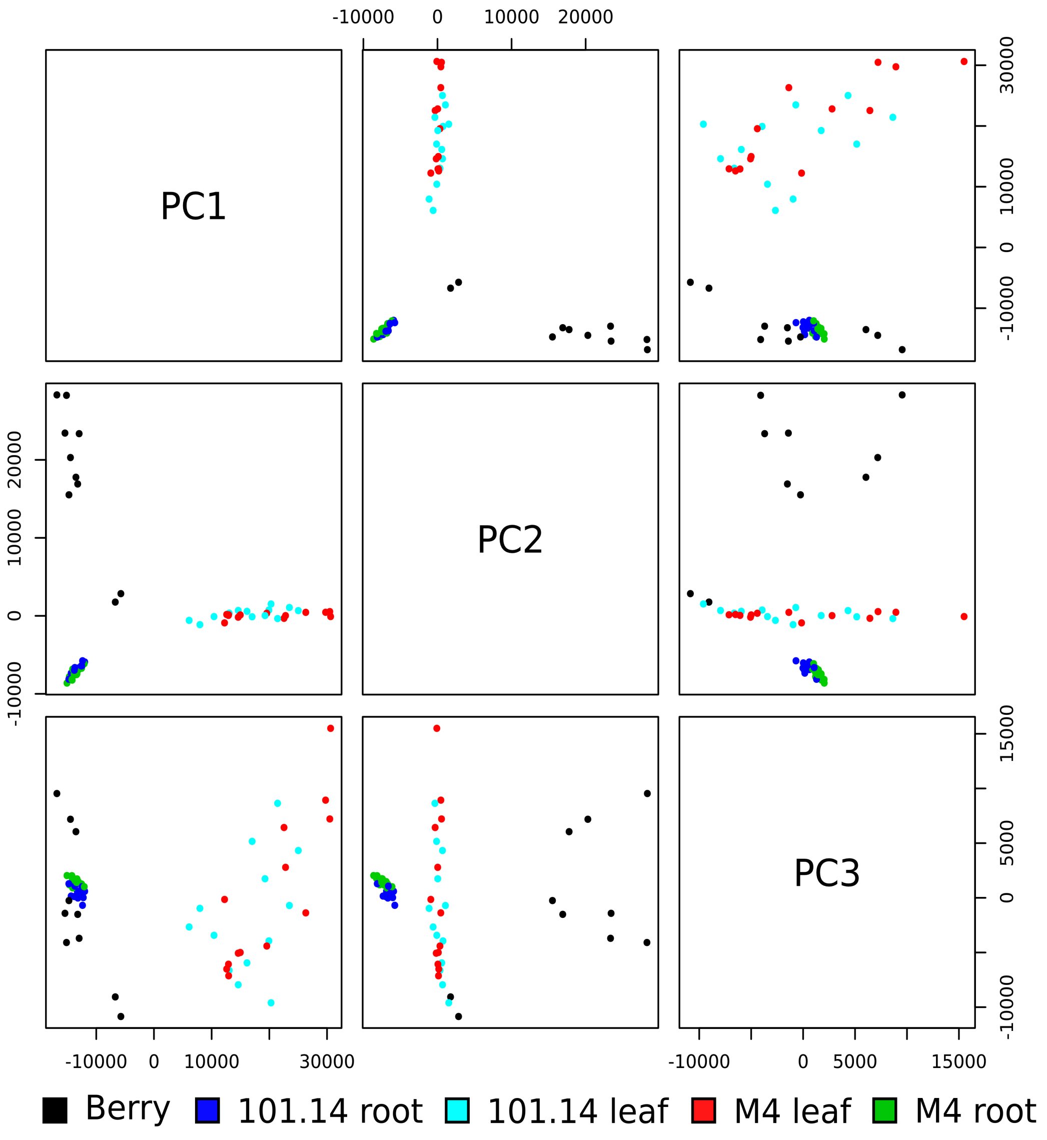

**Supplementary Figure S8: Correlation between lincRNAs structure and expression level.**

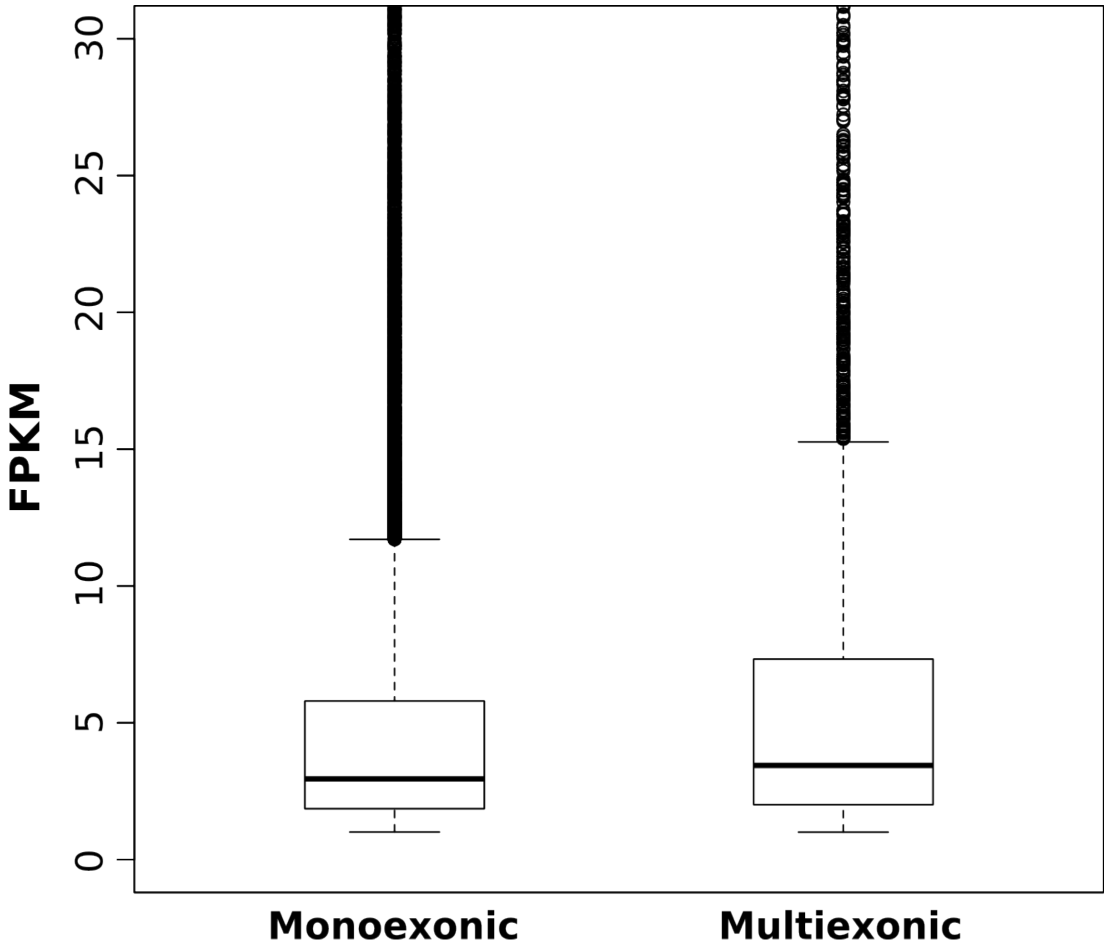

**Splicing factor isoforms and RRM (RNA Recognition Motif) domain.**

Protein domains were identified scanning the longest protein coding sequence against PROSITE database (http://prosite.expasy.org/). The analysis was restricted considering only the isoforms that were differentially expressed between cultivars (highlighted in yellow). Red bars show which part of the transcript encode the RRM (RNA Recognition Motif) domain. The genomic coordinates of the protein domain are reported between bracket. Grey blocks represents UTRs regions while red blocks the coding sequences.

**Supplementary Figure S9: VIT_215s0048g01870**

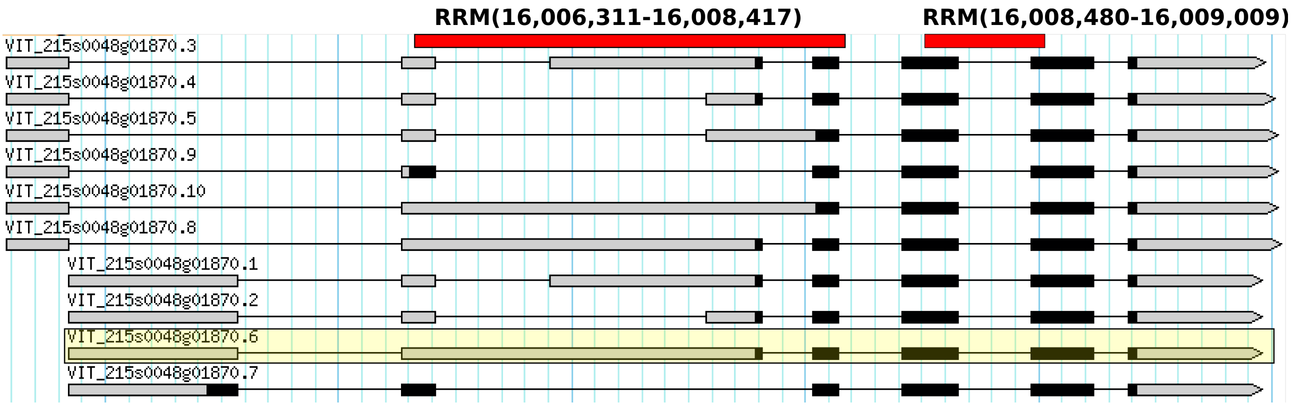


**Supplementary Figure S10: VIT_2160100g00450**

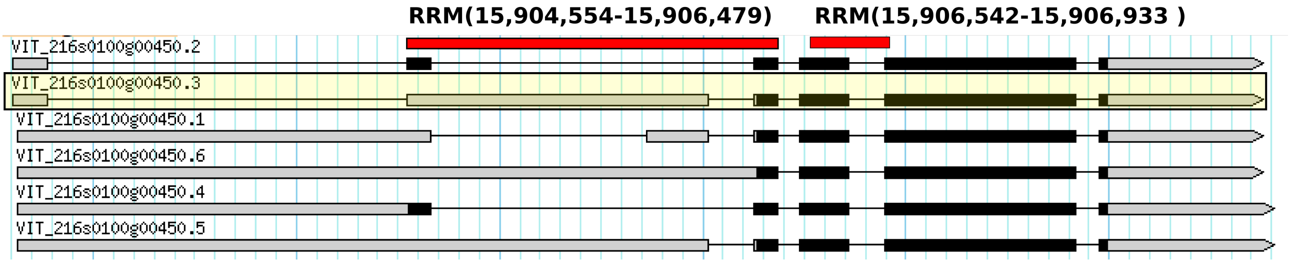

## Supplementary Figure S11: VIT_204s0069g00800

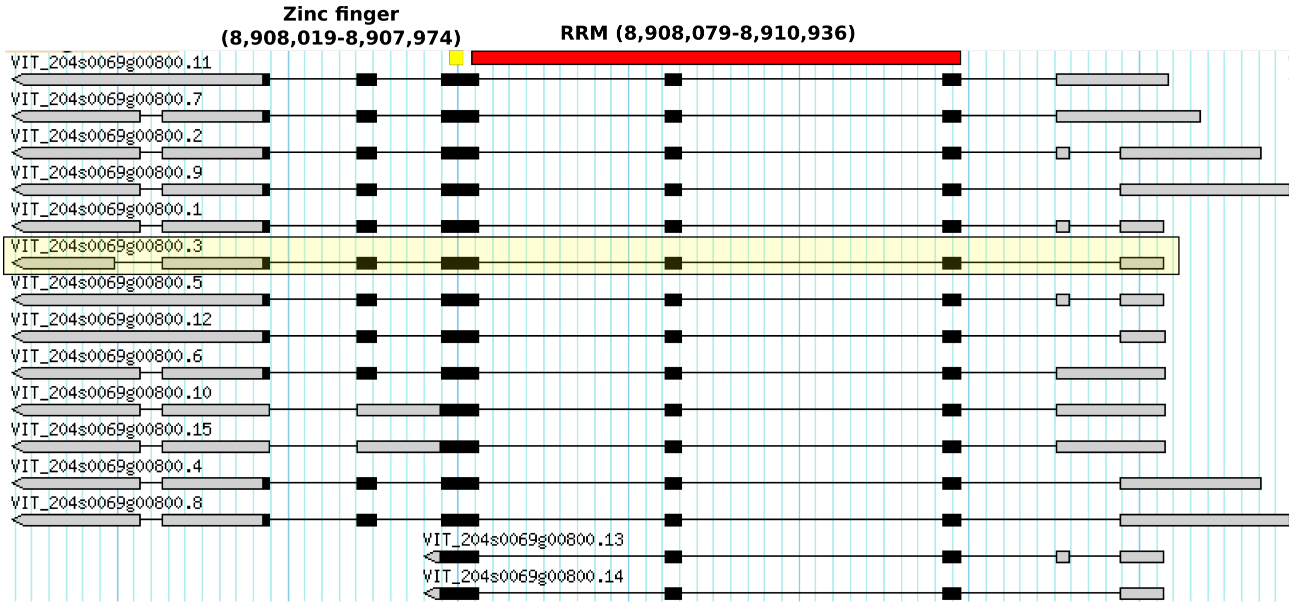


## Supplementary Figure S12: VIT_2160098g01020

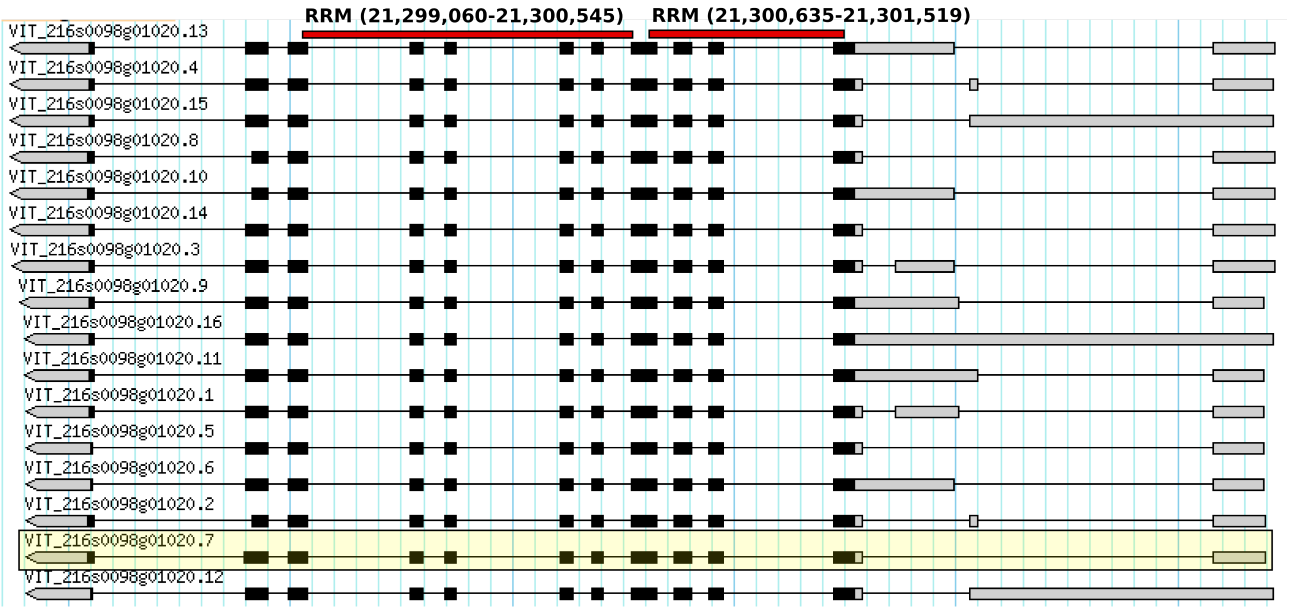

**Supplementary Table S1: Introns support analysis.**

| | All Introns | | Shared | Esclusive Introns | |
|---|---|---|---|---|---|
| | V1 | V2 | V1 and V2 | V1 | V2 |
| # Tot Introns | 117835 | 124401 | 115166 | 5162 | 9235 |
| # confirmed | 105033 | 113685 | 105867 | 2383 | 7818 |
| % confirmed | 89.14 | 91.39 | 91.93 | 46.16 | 84.66 |
| 1 evidence | 12823 (12.2) | 19135 (16.8) | 13848 (13.1) | 1154 (48.4) | 5287 (67.6) |
| 2 evidence | 30434 (29) | 32392 (28.5) | 30584 (28.9) | 564 (23.7) | 1808 (23.1) |
| 3 evidence | 61776 (58.8) | 62158 (54.7) | 61435 (58) | 665 (27.9) | 723 (9.2) |

**Supplementary Table S2: Types and number of evidence confirming introns**

On each cell it is reported the number of sintrons confirmed by that kind of evidence. Label "# evidence" indicates the number of different evidence that simultaneously confirm an intron.

| V1 | | | | | | |
|---|---|---|---|---|---|---|
| | All Introns | | | Esclusive Introns | | |
| # evidence | EST | Protein | RNA-seq | EST | Protein | RNA-seq |
| 1 evidence | 455 | 8594 | 3774 | 46 | 952 | 156 |
| 2 evidence | 11722 | 22029 | 27117 | 239 | 454 | 435 |
| 3 evidence | 61776 | 61776 | 61776 | 665 | 665 | 665 |

| V2 | | | | | | |
|---|---|---|---|---|---|---|
| | All Introns | | | Esclusive Introns | | |
| # evidence | EST | Protein | RNA-seq | EST | Protein | RNA-seq |
| 1 evidence | 404 | 7710 | 11021 | 4 | 186 | 5097 |
| 2 evidence | 12801 | 22790 | 29193 | 854 | 987 | 1775 |
| 3 evidence | 62158 | 62158 | 62158 | 723 | 723 | 723 |

| Shared Introns | | | |
|---|---|---|---|
| # evidence | EST | Protein | RNA-seq |
| 1 evidence | 400 | 7524 | 5924 |
| 2 evidence | 11947 | 21803 | 27418 |
| 3 evidence | 61435 | 61435 | 61435 |

**Supplementary Table S3: Fusion/splitting events analysis**

"**No hit**": the gene models from both the prediction do not have an Arabidopsis homologous protein, "v**1 no hit**" and "v**2 no hit**": v1 or v2 gene model do not have an Arabidopsis homologous protein. "**Ambiguous**": cases where one of the splitted genes do not have a Arabidopisis, homologous sequence. "**v1 optimal**" and "**v2 optimal**": v1 or v2 gene model has a better structure when aligned on Arabidopisis homologous protein.

| | Splitted | Fused |
|---|---|---|
| **No hit** | 5 | 5 |
| **V1 no hit** | 9 | 1 |
| **V2 no hit** | 0 | 0 |
| **Ambiguous** | 10 | 28 |
| **V1 optimal** | 29 | 54 |
| **V2 optimal** | 38 | 161 |
| **Tot** | 91 | 249 |

**Supplementary Table S4: Analysis of the coding-protein genes with a different structure.**

| | |
|---|---|
| **Equal score** | 13 |
| **V2 non homology** | 38 |
| **V1 non homology** | 39 |
| **Both No Homology** | 169 |
| **Different Best Hit** | 458 |
| **Low identity (<30%)** | 610 |
| **V1 better score** | 1432 |
| **V2 better score** | 2207 |
| **Total** | 4966 |

**Splicing factor T-test result tables.**

Significant adjusted p-value (<0.05) are highlighted in red.

**Supplementary Table S5: analysis performed considering the gene expression level**

| Gene | Cultivar | Tissue | Stress |
|---|---|---|---|
| VIT_214s0060g02290 | 5,57E-001 | 2,18E-001 | 9,42E-002 |
| VIT_208s0040g02860 | 1,00E+000 | 5,13E-012 | 1,00E+000 |
| VIT_216s0098g01020 | 1,00E+000 | 6,87E-007 | 2,05E-001 |
| VIT_208s0007g00970 | 1,00E+000 | 5,65E-017 | 1,00E+000 |
| VIT_212s0142g00110 | 1,15E-002 | 7,60E-002 | 9,42E-002 |
| VIT_215s0048g01870 | 1,00E+000 | 3,36E-021 | 1,00E+000 |
| VIT_215s0046g00050 | 5,59E-002 | 6,03E-001 | 7,50E-004 |
| VIT_219s0027g00590 | 1,00E+000 | 6,83E-023 | 1,00E+000 |
| VIT_213s0156g00020 | 1,00E+000 | 1,89E-024 | 1,00E+000 |
| VIT_201s0026g00250 | 1,00E+000 | 2,62E-008 | 2,12E-002 |
| VIT_206s0004g00710 | 1,00E+000 | 2,59E-015 | 1,00E+000 |
| VIT_218s0001g05550 | 1,00E+000 | 6,40E-009 | 1,25E-001 |
| VIT_213s0067g03600 | 1,00E+000 | 6,56E-034 | 1,00E+000 |
| VIT_207s0005g00320 | 1,00E+000 | 9,57E-001 | 1,00E+000 |
| VIT_213s0019g01060 | 1,00E+000 | 2,52E-020 | 1,00E+000 |
| VIT_214s0030g00480 | 6,14E-001 | 3,45E-009 | 1,00E+000 |
| VIT_204s0069g00800 | 1,00E+000 | 2,24E-016 | 1,00E+000 |
| VIT_216s0100g00450 | 1,00E+000 | 1,59E-002 | 1,00E+000 |

**Supplementary Table S6**: analysis performed considering the transcript expression level

| Transcript ID | Cultivar | Tissue | Stress |
|---|---|---|---|
| VIT_201s0026g00250.1 | 1,00E+000 | 1,66E-001 | 1 |
| VIT_201s0026g00250.2 | 1,00E+000 | 1,49E-003 | 0,5777426693 |
| VIT_204s0069g00800.1 | 1,00E+000 | 2,30E-013 | 1 |
| VIT_204s0069g00800.10 | 4,39E-001 | 7,23E-004 | 1 |
| VIT_204s0069g00800.12 | 1,00E+000 | 1,46E-014 | 1 |
| VIT_204s0069g00800.3 | 4,79E-005 | 9,83E-001 | 1 |
| VIT_204s0069g00800.5 | 1,00E+000 | 1,57E-001 | 1 |
| VIT_206s0004g00710.1 | 1,00E+000 | 1,01E-014 | 1 |
| VIT_207s0005g00320.1 | 1,00E+000 | 1,00E+000 | 1 |
| VIT_208s0007g00970.1 | 1,00E+000 | 1,91E-016 | 1 |
| VIT_208s0040g02860.1 | 1,00E+000 | 2,00E-011 | 1 |
| VIT_212s0142g00110.1 | 2,88E-002 | 2,85E-001 | 0,2649890248 |
| VIT_213s0019g01060.1 | 1,00E+000 | 8,11E-020 | 1 |
| VIT_213s0067g03600.1 | 1,00E+000 | 1,93E-004 | 1 |
| VIT_213s0067g03600.2 | 1,00E+000 | 5,15E-030 | 1 |
| VIT_213s0156g00020.1 | 1,00E+000 | 5,22E-024 | 1 |
| VIT_214s0030g00480.1 | 1,00E+000 | 1,34E-008 | 1 |
| VIT_214s0060g02290.2 | 6,58E-001 | 1,00E+000 | 0,2345609883 |
| VIT_214s0060g02290.3 | 1,00E+000 | 1,00E+000 | 1 |
| VIT_215s0046g00050.1 | 8,33E-001 | 1,00E-001 | 1 |
| VIT_215s0046g00050.2 | 6,80E-001 | 5,26E-008 | 1 |
| VIT_215s0046g00050.3 | 1,00E+000 | 4,66E-003 | 1 |
| VIT_215s0046g00050.4 | 1,88E-001 | 2,53E-001 | 0,0766044587 |
| VIT_215s0048g01870.10 | 1,00E+000 | 5,29E-010 | 1 |
| VIT_215s0048g01870.3 | 1,00E+000 | 1,60E-002 | 1 |
| VIT_215s0048g01870.4 | 1,00E+000 | 4,08E-011 | 1 |
| VIT_215s0048g01870.6 | 2,87E-003 | 1,00E+000 | 1 |
| VIT_215s0048g01870.8 | 7,46E-002 | 1,66E-001 | 1 |
| VIT_215s0048g01870.9 | 1,00E+000 | 3,59E-013 | 1 |
| VIT_216s0098g01020.1 | 1,46E-001 | 1,00E+000 | 1 |
| VIT_216s0098g01020.11 | 1,00E+000 | 1,00E+000 | 1 |
| VIT_216s0098g01020.13 | 1,00E+000 | 1,46E-002 | 1 |
| VIT_216s0098g01020.14 | 1,00E+000 | 2,20E-006 | 1 |
| VIT_216s0098g01020.15 | 1,00E+000 | 1,00E+000 | 1 |
| VIT_216s0098g01020.2 | 1,00E+000 | 6,61E-001 | 1 |
| VIT_216s0098g01020.3 | 1,00E+000 | 1,00E+000 | 1 |
| VIT_216s0098g01020.4 | 1,00E+000 | 7,35E-004 | 1 |
| VIT_216s0098g01020.5 | 1,00E+000 | 3,24E-002 | 1 |
| VIT_216s0098g01020.6 | 1,00E+000 | 1,00E+000 | 1 |
| VIT_216s0098g01020.7 | 3,73E-002 | 1,00E+000 | 1 |
| VIT_216s0098g01020.9 | 1,88E-001 | 1,00E+000 | 1 |
| VIT_216s0100g00450.2 | 1,00E+000 | 6,19E-003 | 0,9464053289 |
| VIT_216s0100g00450.3 | 7,06E-004 | 1,00E+000 | 1 |
| VIT_216s0100g00450.6 | 1,00E+000 | 1,79E-004 | 1 |
| VIT_218s0001g05550.1 | 1,00E+000 | 6,44E-009 | 1 |
| VIT_218s0001g05550.2 | 1,00E+000 | 4,17E-004 | 1 |
| VIT_218s0001g05550.3 | 1,00E+000 | 1,57E-001 | 0,061900118 |
| VIT_219s0027g00590.1 | 1,00E+000 | 1,96E-022 | 1 |
